# Antibiotic De-Escalation in Adults Hospitalized for Community-Onset Sepsis

**DOI:** 10.1001/jamainternmed.2025.6919

**Published:** 2025-12-22

**Authors:** Ashwin B. Gupta, Megan Heath, Emily Walzl, David Ratz, Elizabeth Munroe, Valerie M. Vaughn, Jennifer K. Horowitz, Tawny Czilok, Lindsay A. Petty, Tejal N. Gandhi, Stephanie Parks Taylor, Elizabeth S. McLaughlin, Patricia Posa, Anurag N. Malani, Lama Hsaiky, Scott A. Flanders, Hallie C. Prescott

**Affiliations:** 1 Medicine Service, VA Ann Arbor Healthcare System, Ann Arbor, Michigan; 2 Department of Internal Medicine, University of Michigan, Ann Arbor; 3 VA Center for Clinical Management Research, Ann Arbor, Michigan; 4 Department of Internal Medicine, University of Utah, Salt Lake City; 5 Division of Health System Innovation & Research, Department of Population Health Sciences, University of Utah, Salt Lake City; 6 Section of Infectious Diseases, Trinity Health Michigan, Ann Arbor; 7 Department of Pharmacy, Corewell Healthcare System, Dearborn, Michigan

## Abstract

**Importance:**

Prolonged exposure to broad-spectrum antibiotics (BSA) may be associated with patient harm.

**Objective:**

To assess outcomes of BSA de-escalation compared with continuation on encounter day 4 in patients hospitalized for community-onset sepsis.

**Design, Setting, and Participants:**

This target trial emulation study was conducted at 67 hospitals participating in the Michigan Hospital Medicine Safety Consortium. Patients 18 years and older hospitalized for community-onset sepsis from June 2020 through September 2024 who initiated empiric BSA therapy without evidence of multidrug-resistant organism infection were included. Data were analyzed from September 2024 to November 2025.

**Exposure:**

Inverse probability of treatment–weighted cohort exposed to de-escalation of BSA vs continuation at day 4.

**Main Outcomes and Measures:**

The primary outcome was 90-day all-cause mortality. Secondary outcomes included in-hospital mortality, 30-day mortality, length of hospitalization, and days of antibiotic therapy.

**Results:**

Among 36 924 patients with community-onset sepsis, 18 559 (50.3%) were female, 18 365 (49.7%) were male, and the median (IQR) age was 71 (61-80) years. A total of 6926 (18.8%) and 11 149 (30.2%) were eligible for target trial emulations evaluating de-escalation of anti–methicillin-resistant *Staphylococcus aureus* (MRSA) and anti–*Pseudomonas aeruginosa* (PSA; or other resistant gram-negative bacteria) antibiotics, respectively. Of these, 2993 (43.2%) and 2493 (22.4%) were de-escalated from anti-MRSA and anti-PSA coverage. After weighting, patients who were de-escalated vs continued on BSA were well balanced on baseline characteristics. In weighted analyses, anti-MRSA and anti-PSA de-escalation were associated with similar 90-day mortality as continued BSA therapy (anti-MRSA: odds ratio, 1.00; 95% CI, 0.88-1.14; anti-PSA: odds ratio, 0.98; 95% CI, 0.86-1.13). Additionally, anti-MRSA and anti-PSA de-escalation were associated with fewer days of antibiotics to day 14 (anti-MRSA de-escalation: risk ratio [RR], 0.91; 95% CI, 0.89-0.93; anti-PSA de-escalation: RR, 0.91; 95% CI, 0.88-0.93) and shorter length of hospitalization (anti-MRSA de-escalation: RR, 0.88; 95% CI, 0.85-0.92; anti-PSA de-escalation: RR, 0.91; 0.88-0.93). All other secondary outcomes were similar. Across 67 hospitals, the proportion of eligible patients de-escalated from BSA varied more than 2-fold (anti-MRSA de-escalation, 27.3% to 61.7%; anti-PSA de-escalation, 6.9% to 37.7%).

**Conclusions and Relevance:**

In this study, de-escalation of empiric BSA therapy at day 4 was associated with similar safety outcomes, fewer antibiotic days, and shorter length of hospitalization among patients with community-onset sepsis compared with those who continued BSA therapy but varied widely in practice.

## Key Points

**Question:**

Is broad-spectrum antibiotic de-escalation on encounter day 4 safe among patients hospitalized with community-onset sepsis who have no positive test result for multidrug-resistant organisms (MDROs)?

**Findings:**

In this target trial emulation study including 36 924 patients with community-onset sepsis, de-escalation from methicillin-resistant *Staphylococcus aureus* (MRSA) or *Pseudomonas aeruginosa* coverage were associated with similar 90-day all-cause mortality, fewer days of antibiotic therapy, and shorter length of hospitalizations compared with continued broad-spectrum coverage.

**Meaning:**

These results suggest that de-escalation of broad-spectrum antibiotics among patients with community-onset sepsis and no positive MDRO test results is associated with similar safety outcomes and may reduce antibiotic exposure and length of hospitalization.

## Teaser

This target trial emulation study assesses outcomes of broad-spectrum antibiotics de-escalation compared with continuation on encounter day 4 in patients hospitalized for community-onset sepsis.

## Introduction

Early empiric broad-spectrum antibiotic (BSA) therapy covering multidrug-resistant organisms (MDROs) has been associated with lower mortality in patients at heightened risk of MDRO infection and is recommended by the 2021 Surviving Sepsis Campaign Guidelines.^1,2,3,4,5,6,7^ While empiric BSAs are an important component of sepsis management, their use is associated with increased risk for antibiotic-associated adverse events,^8,9,10^ including *Clostridioides difficile* infection, and development of population-level antimicrobial resistance.^11,12,13^ Furthermore, a recent study assessing empiric coverage of methicillin-resistant *Staphylococcus aureus* (MRSA) in patients hospitalized for pneumonia found no mortality benefit for any patient subgroup examined, even those with risk factors for MRSA.^14^ Timely de-escalation of empiric BSA therapy is thus recommended if MDROs are not identified on initial testing.^6,7,15,16,17^ The incidence of MDRO positivity among patients with community-onset sepsis varies across settings, although several US cohorts suggest MDROs are isolated in approximately 10% of sepsis hospitalizations.^18,19,20^

Despite guideline recommendations to de-escalate antibiotics, the incidence of antibiotic de-escalation and impact on clinical outcomes in sepsis remain unclear. Studies evaluating BSA de-escalation vs continued BSA use in patients with sepsis have yielded mixed results, with some suggesting de-escalation improves clinical cure^21^ and survival^22,23^ but others finding no impact on mortality and other unintended consequences, such as more secondary infections and more days of antibiotic therapy.^24^ As a result, the clinical implications of antibiotic de-escalation in patients with sepsis remain unclear.

In this study, we sought to evaluate patient outcomes associated with antibiotic de-escalation in the absence of positive findings on MDRO culture and examine the variability in antibiotic de-escalation practices for adult patients hospitalized with community-onset sepsis across Michigan hospitals.

## Methods

### Study Setting and Design

We used data from the Michigan Hospital Medicine Safety Consortium (HMS) Sepsis Initiative (HMS-Sepsis) to conduct 2 target trial emulation studies of BSA de-escalation. HMS is a multihospital quality improvement collaborative in Michigan. Additional information about HMS is provided in eAppendix 1 in Supplement 1.

This study was deemed not regulated by the University of Michigan Institutional Review Board because HMS is a quality improvement initiative. We followed Strengthening the Reporting of Observational Studies in Epidemiology (STROBE) reporting guideline.

### HMS-Sepsis Registry

The HMS-Sepsis registry includes data on a random sample of community-onset sepsis hospitalizations entered by professional abstractors at each participating hospital. Details on sampling, data collection, and data validation have been described previously.^25^ Briefly, qualifying hospitalizations are identified via a 2-step process. First, hospitalizations with a principal diagnosis of sepsis, infection, or respiratory failure (with secondary diagnosis of pneumonia) are identified. Then, abstractors review the health record to confirm presence of infection and acute organ dysfunction during the first 2 days of the encounter. Specifically, abstractors assess for lactate elevation, acute respiratory dysfunction, acutely altered mental status, acute kidney dysfunction, acute hematologic dysfunction, acute liver dysfunction, and vasopressor treatment, similar to the US Centers for Disease Control and Prevention Adult Sepsis Event criteria.^26^ This 2-step process ensures consistent identification of sepsis despite variable diagnostic coding.^27^ Data on antibiotics administered from presentation through day 14 are captured in the HMS-Sepsis registry, including antibiotics prescribed at hospital discharge. Additional information about the HMS-Sepsis registry is provided in eAppendix 2 in Supplement 1.

### Study Cohort and Exposures

This study was performed as 2 target trial emulations^28^ of BSA de-escalation: one assessing de-escalation of anti-MRSA antibiotics and the other assessing de-escalation of anti-pseudomonal antibiotics. We sought to understand outcomes associated with de-escalation of these BSAs at hospital day 4 in patients hospitalized for community-onset sepsis. The target trial protocol is presented in eTable 1 in Supplement 1. We included patients hospitalized with community-onset sepsis (hospitalized June 2020 to August 2024; discharged November 2020 through September 2024) who initiated BSAs and had no evidence of MDRO positivity on encounter days 1 or 2 (either negative findings for MDRO or cultures not collected). Time 0 (time of enrollment and randomization in the target trial) was defined as the end of the third calendar day of the hospitalization encounter, which included time in the emergency department.

The specific BSA therapies of interest were antibiotic therapy targeting MRSA or resistant gram-negative bacteria on encounter day 1 (day of emergency department presentation) or day 2 and continued on day 3. Resistant gram-negative bacteria of interest were *Pseudomonas aeruginosa* (PSA), *Achromobacter*, *Acinetobacter*, *Citrobacter*, *Enterobacter*, *Morganella*, and *Serratia*—collectively referred to as PSA. Qualifying anti-MRSA and anti-PSA antibiotics are listed in eTable 2 in Supplement 1. MDRO positivity was assessed based on cultures of blood, bronchoalveolar lavage fluid, cerebrospinal fluid, endotracheal aspirate, peritoneal fluid, pleural fluid, sputum, upper respiratory/nasopharyngeal swabs, and urine, as well as MRSA nares swabs. We considered only testing obtained on day 1 or day 2 for eligibility, as day 3 testing would not have resulted at time of enrollment. Patients who initiated anti*-*MRSA and anti*-*PSA antibiotics were analyzed separately, and only patients alive through encounter day 3 were included to avoid immortal time bias.

We compared patients receiving BSA de-escalation (ie, no BSA on day 4) with those receiving continued BSA therapy (ie, BSA on day 4). Under the intention-to-treat principle, subsequent antibiotic treatment was not considered in treatment assignment. We used a 3-day cutoff for BSA de-escalation since guidelines recommend reassessing empiric antimicrobials within 48 to 72 hours.^15,17^

### Primary and Secondary Outcomes

The primary outcome was 90-day mortality from enrollment into the target trial. We selected this time point since antibiotic selection and duration may impact longer-term but not shorter-term mortality.^29^ Secondary outcomes were in-hospital mortality, 30-day mortality, composite of in-hospital mortality or hospice discharge, length of hospitalization, and days of antibiotic therapy to day 14, inclusive of antibiotics prescribed at hospital discharge. Exploratory outcomes were 90-day all-cause rehospitalization (among patients discharged alive, not transferred to another hospital, and not discharged to hospice) and *C difficile* infection at 90 days. Mortality outcomes were captured via medical record review, public obituary data, and follow-up by telephone, email, and/or SMS messaging. Readmission and *C difficile* infection were captured via medical record review. Data from other hospitals are variably available to the discharging hospital, so readmission and *C difficile* infection were considered exploratory outcomes.

### Statistical Analysis

To control for confounding, we used inverse probability of treatment weighting to balance the de-escalated and continued BSA therapy groups on likelihood of being de-escalated. The propensity score model included both patient characteristics and a fixed effect for each hospital. Before proceeding to analysis, we assessed both the propensity score distribution and the balance of patient characteristics and treating hospital to ensure good balance (defined as a standardized mean difference [SMD] less than 0.10).^30,31,32^ Baseline patient characteristics in the propensity model included demographic characteristics; chronic health conditions; early hospitalization data, including site of infection and predicted 30-day mortality from the HMS-Sepsis mortality model^25^; physiology at time of enrollment and randomization, including vital signs, laboratory values, and level of care; cultures collected; and antibiotic cotreatment. The full list of variables used in weighting, their functional form in the propensity score model, and process for imputing missing data are presented in eTable 3 in Supplement 1. Site of infection was determined from *International Statistical Classification of Diseases and Related Health Problems, Tenth Revision *discharge diagnosis codes, categorized using Healthcare Cost and Utilization Project’s Clinical Classifications Software Refined,^33^ as shown in eTable 4 in Supplement 1. A specified site of infection was identified for 83.6% of hospitalizations. Site of infection was included with the target trial protocol because, while measured via discharge diagnosis coding, it is generally known by day 3 in clinical practice, so it likely reflected clinician knowledge time of enrollment into the target trial. We next fit a series of hierarchical logistic and Poisson regression models (with hospitalizations nested within hospitals) to assess odds ratios (ORs) and risk ratios (RRs), respectively, for binary and count outcomes. We fit separate models for each outcome and exposure pairing. Second, we examined variation in de-escalation among eligible patients across hospitals.

Analyses were performed in SAS version 9.4 (SAS Institute) from September 2024 to November 2025. Given possible disparities by patient demographic characteristic, we report data on sex and self-reported race and ethnicity obtained from the health record using HMS definitions, detailed in eAppendix 3 in Supplement 1. We considered 2-tailed *P* < .05 as statistically significant and calculated E-values^34,35^ to estimate the strength of association of a hypothetical unmeasured confounder that would be needed to negate the measured associations between antibiotic de-escalation and outcomes.

We completed a subgroup analysis among patients with clinical stability at enrollment (day 3), as patient stability likely impacts a clinician’s decision to de-escalate antibiotics. Similar to a prior study of antibiotic de-escalation,^36^ clinical stability was defined as not receiving vasopressor therapy; not receiving invasive mechanical ventilation; and having no more than 1 abnormal vital sign (temperature greater than 38 °C, oxygen saturation less than 90%, supplemental oxygen therapy above prehospital baseline, heart rate greater than 100 beats per minute, respiratory rate greater than 24 breaths per minute, or systolic blood pressure less than 90 mm Hg).

## Results

### Primary Cohort

#### Anti-MRSA De-Escalation

**Figure 1.**
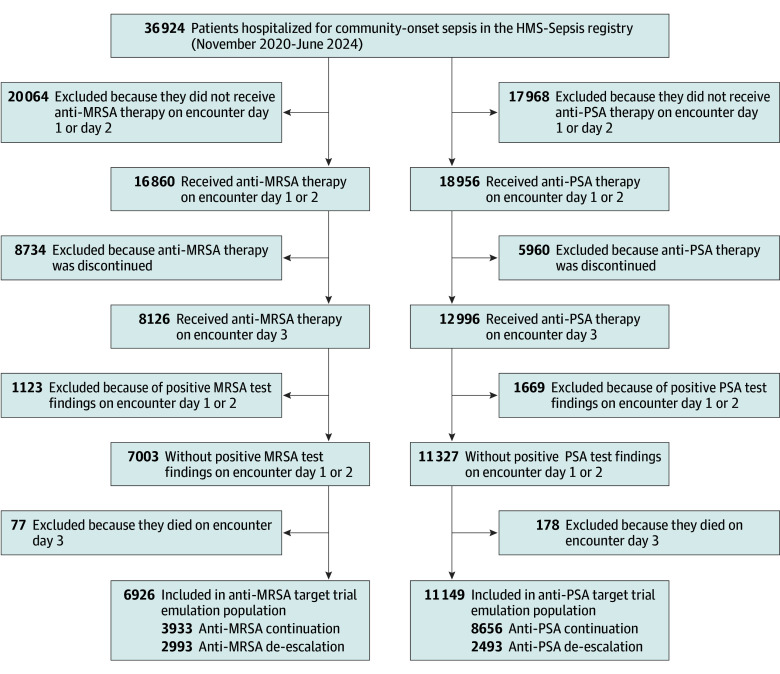
CONSORT Diagram Among 6923 patients in the anti–methicillin-resistant *Staphylococcus aureus* (MRSA) de-escalation target trial emulation, 2161 were clinically stable on day 3, including 891 who were de-escalated and 1270 who were continued on anti-MRSA therapy. Among 11 149 patients in the anti–*Pseudomonas aeruginosa* (PSA; or other resistant gram-negative bacteria) target trial emulation, 3344 were clinically stable on day 3, including 837 who were de-escalated and 2507 who were continued on anti-PSA therapy. HMS-Sepsis indicates Michigan Hospital Medicine Safety Consortium Sepsis Initiative.

Among 36 924 patients hospitalized with community-onset sepsis during the study period, 18 559 (50.3%) were female, 18 365 (49.7%) were male, and the median (IQR) age was 71 (61-80) years. A total of 5671 patients (15.4%) were Black, 29 336 (79.5%) were White, 1024 (2.8%) were other race (including American Indian or Alaskan Native race, Arab and Chaldean ancestry, Asian race, Native Hawaiian or Other Pacific Islander race, and other race), and 893 (2.4%) had unknown race; 751 (2.0%) were Hispanic/Latino, 28 124 (76.2%) were non-Hispanic, and 8049 (21.8%) had unknown ethnicity. A total of 16 860 (45.7%) initiated anti-MRSA antibiotics on encounter days 1 or 2, of which 8126 (48.2%) continued taking anti-MRSA antibiotics on day 3 (Figure 1). Of these, 1123 had positive MRSA test findings and 77 died on day 3, yielding 6926 patients for enrollment into the target trial of anti-MRSA de-escalation. Of 6926 patients in the target trial of anti-MRSA de-escalation, 3347 (48.3%) had cultures positive for other organisms, 3461 (50.0%) had negative culture findings, and 118 (1.7%) had no cultures obtained on encounter days 1 and 2.

A total of 2993 patients (43.2%) in the target trial of anti-MRSA de-escalation were de-escalated on day 4. Prior to weighting, the de-escalated and continued BSA groups were unbalanced on several patient characteristics and treating hospital, as shown in eTables 5 and 6 and eFigure 1 in Supplement 1. De-escalated patients had a median (IQR) age of 70 (59-78) years and 1623 (57.7%) were male; patients who continued BSA therapy had a median (IQR) age 68 (57-77) years and 2186 (58.8%) were male (eTable 5 in Supplement 1). The most imbalanced characteristics in the anti-MRSA target trial were anti-PSA de-escalation, MRSA swab collection, and creatinine. After weighting, the de-escalated and continued BSA therapy groups were well balanced on propensity scores, patient characteristics (SMD less than 0.10 for all), and treating hospital (Table 1; eFigures 1 and 2 and eTables 5 and 7 in Supplement 1).

**Table 1.** Characteristics of Target Trial Emulation Patients After Weighting

Characteristic	Coverage cohort, No. (%)					
	Anti-MRSA			Anti-PSA		
	De-escalated (n = 2993)	Continued (n = 3933)	SMD	De-escalated (n = 2493)	Continued (n = 8656)	SMD
Demographic characteristics						
Age, median (IQR), y	69 (58 to 77)	68 (58 to 77)	−0.01	70 (60 to 78)	70 (60 to 79)	−0.02
Sex						
Female	1263 (42.2)	1707 (43.4)	0.02	1159 (46.5)	3843 (44.4)	−0.04
Male	1730 (57.8)	2226 (56.6)		1334 (53.5)	4813 (55.6)	
Self-reported race						
Black	518 (17.3)	673 (17.1)	0.01	409 (16.4)	1420 (16.4)	0
White	1 [Reference]	1 [Reference]	NA	1 [Reference]	1 [Reference]	NA
Other or unknown race^a^	84 (2.8)	110 (2.8)	0	82 (3.3)	251 (2.9)	0.03
Baseline health information						
BMI, median (IQR)^b^	28.8 (23.1 to 35.5)	28.6 (23.2 to 35.5)	−0.01	28.0 (22.8 to 34.2)	27.8 (22.7 to 34.0)	0.01
Charlson Comorbidity Index score, median (IQR)	3 (2 to 5)	3 (1 to 5)	0	3 (2 to 5)	3 (2 to 5)	0.01
Functional limitations, median (IQR)	0 (0 to 5)	0 (0 to 5)	0	0 (0 to 5)	0 (0 to 5)	−0.02
Hospitalization in prior 90 d	1230 (41.1)	1585 (40.3)	0.02	1119 (44.9)	3765 (43.5)	0.03
Admitted from skilled nursing facility, subacute rehabilitation, or long-term acute care	500 (16.7)	653 (16.6)	0	464 (18.6)	1515 (17.5)	0.03
Moderate or severe kidney disease	913 (30.5)	1235 (31.4)	−0.02	912 (36.6)	3056 (35.3)	0.03
Hypertension	2029 (67.8)	2702 (68.7)	−0.02	1735 (69.6)	5981 (69.1)	0.01
Peripheral vascular disease	464 (15.5)	586 (14.9)	0.02	356 (14.3)	1203 (13.9)	0.01
Cardiovascular disease	1230 (41.1)	1613 (41.0)	0	1065 (42.7)	3636 (42.0)	0.02
Atrial fibrillation	694 (23.2)	932 (23.7)	−0.01	626 (25.1)	2147 (24.8)	0.01
Congestive heart failure	778 (26.0)	1034 (26.3)	−0.01	725 (29.1)	2372 (27.4)	0.04
Leukemia or lymphoma	120 (4.0)	153 (3.9)	0	105 (4.2)	424 (4.9)	−0.03
Metastatic solid tumor	302 (10.1)	393 (10.0)	0	264 (10.6)	952 (11.0)	−0.01
Cognitive impairment	572 (19.1)	755 (19.2)	0	476 (19.1)	1697 (19.6)	−0.01
Moderate or severe liver disease	96 (3.2)	118 (3.0)	0.01	82 (3.3)	286 (3.3)	0
Mild liver disease	153 (5.1)	208 (5.3)	−0.01	150 (6.0)	485 (5.6)	0.02
Early hospitalization data (pre-enrollment into the target trial)						
Site of infection						
Bacteremia	3 (0.1)	0	0.04	2 (0.1)	9 (0.1)	0
Cardiac	33 (1.1)	51 (1.3)	−0.02	17 (0.7)	43 (0.5)	0.02
Central nervous system	48 (1.6)	67 (1.7)	−0.01	20 (0.8)	52 (0.6)	0.02
Gastrointestinal	174 (5.8)	236 (6.0)	−0.01	219 (8.8)	770 (8.9)	0
Genitourinary	389 (13.0)	503 (12.8)	0.01	451 (18.1)	1541 (17.8)	0.01
Skin or soft tissue	685 (22.9)	873 (22.2)	0.02	297 (11.9)	1021 (11.8)	0
Respiratory	1185 (39.6)	1597 (40.6)	−0.02	1065 (42.7)	3713 (42.9)	0
Other specified	12 (0.4)	16 (0.4)	0	7 (0.3)	26 (0.3)	0
Not identified	464 (15.5)	594 (15.1)	0.01	414 (16.6)	1489 (17.2)	−0.02
Viral sepsis	147 (4.9)	197 (5.0)	0	150 (6.0)	528 (6.1)	0
Predicted 30-d mortality, median (IQR)	0.13 (0.06 to 0.28)	0.13 (0.05 to 0.29)	0	0.15 (0.06 to 0.32)	0.16 (0.07 to 0.31)	0
Altered mental status	1505 (50.3)	1963 (49.9)	0.01	1239 (49.7)	4233 (48.9)	0.02
Receipt of dialysis	132 (4.4)	173 (4.4)	0	184 (7.4)	537 (6.2)	0.05
Receipt of mechanical ventilation	455 (15.2)	606 (15.4)	0	396 (15.9)	1212 (14)	0.06
Enrollment (day 3) physiology and treatments						
Highest temperature, °C						
<35.0	3 (0.1)	4 (0.1)	0.01	0	0	0
35.0-36.0	9 (0.3)	12 (0.3)	0	12 (0.5)	35 (0.4)	0.02
36.1-37.8	2574 (86.0)	3367 (85.6)	0.01	2137 (85.7)	7470 (86.3)	−0.02
37.9-38.0	90 (3.0)	126 (3.2)	−0.01	75 (3.0)	260 (3.0)	0
38.1-38.3	108 (3.6)	146 (3.7)	−0.01	95 (3.8)	294 (3.4)	0.02
38.4-39.9	195 (6.5)	256 (6.5)	0	157 (6.3)	528 (6.1)	0.01
≥40	18 (0.6)	24 (0.6)	0	17 (0.7)	69 (0.8)	−0.02
Highest heart rate, beats/min						
<60	15 (0.5)	24 (0.6)	−0.01	20 (0.8)	52 (0.6)	0.02
60-90	922 (30.8)	1215 (30.9)	0	768 (30.8)	2761 (31.9)	−0.02
91-100	703 (23.5)	905 (23.0)	0.01	571 (22.9)	1930 (22.3)	0.01
101-124	1000 (33.4)	1318 (33.5)	0	845 (33.9)	2874 (33.2)	0.01
≥125	353 (11.8)	476 (12.1)	−0.01	289 (11.6)	1039 (12.0)	−0.01
Lowest systolic blood pressure, mm Hg						
<90	649 (21.7)	861 (21.9)	−0.01	568 (22.8)	1939 (22.4)	0.01
90-100	626 (20.9)	822 (20.9)	0	541 (21.7)	1835 (21.2)	0.01
≥101	1721 (57.5)	2250 (57.2)	0.01	1384 (55.5)	4882 (56.4)	−0.02
Highest respiratory rate, breaths/min						
<20	868 (29.0)	1148 (29.2)	0	740 (29.7)	2554 (29.5)	0
20-21	599 (20.0)	783 (19.9)	0	471 (18.9)	1671 (19.3)	−0.01
22-24	458 (15.3)	594 (15.1)	0	399 (16.0)	1342 (15.5)	0.01
25-30	530 (17.7)	688 (17.5)	0	426 (17.1)	1463 (16.9)	0
>30	539 (18.0)	720 (18.3)	−0.01	459 (18.4)	1619 (18.7)	−0.01
Lowest Pao_2_/FIO_2_, median (IQR), mm Hg	250.0 (180.6 to 309.5)	250.0 (180.6 to 309.5)	0.02	232.1 (180.6 to 309.5)	232.1 (180.6 to 309.5)	0.03
Maximum respiratory support						
Room air	1 [Reference]	1 [Reference]	NA	1 [Reference]	1 [Reference]	NA
Invasive mechanical ventilation	455 (15.2)	602 (15.3)	0	379 (15.2)	1186 (13.7)	0.04
NIPPV	189 (6.3)	248 (6.3)	0	162 (6.5)	571 (6.6)	0
HFNC	114 (3.8)	157 (4.0)	−0.01	95 (3.8)	355 (4.1)	−0.01
Low-flow oxygen system	1170 (39.1)	1550 (39.4)	−0.01	1027 (41.2)	3636 (42.0)	−0.02
Laboratory values, median (IQR)						
Lowest hemoglobin, g/dL	10.1 (8.6 to 11.7)	10.1 (8.6 to 11.7)	−0.03	10.0 (8.5 to 11.5)	10.0 (8.5 to 11.5)	−0.01
Highest white blood cell count, cells/μL	11.3 (7.7 to 16.2)	11.3 (8.0 to 16.2)	−0.01	11.4 (7.9 to 16.1)	11.3 (7.8 to 16.1)	0.02
Highest lactate, mg/dL	17.1 (10.8 to 24.3)	16.2 (10.8 to 24.3)	0.01	17.1 (10.8 to 24.3)	16.2 (10.8 to 24.4)	−0.01
Lowest platelet count, ×10^3^/μL	0.20 (0.14 to 0.27)	0.20 (0.14 to 0.27)	0	0.20 (0.13 to 0.27)	0.19 (0.13 to 0.27)	0
Highest creatinine, mg/dL	0.9 (0.7 to 1.4)	0.9 (0.7 to 1.4)	0.01	1.1 (0.8 to 1.7)	1.0 (0.7 to 1.7)	0.03
Highest bilirubin, mg/dL	0.6 (0.4 to 1.0)	0.6 (0.4 to 1.0)	0.01	0.6 (0.4 to 1.0)	0.7 (0.4 to 1.0)	0
Highest procalcitonin, μg/L	0.1 (0.1 to 0.7)	0.1 (0.1 to 0.8)	−0.04	0.1 (0.1 to 0.9)	0.1 (0.1 to 0.9)	0.01
Vasopressors administered	458 (15.3)	610 (15.5)	−0.01	394 (15.8)	1350 (15.6)	0.01
Highest level of care						
ICU	1045 (34.9)	1373 (34.9)	0	947 (38.0)	3082 (35.6)	0.05
Step-down	715 (23.9)	924 (23.5)	−0.01	563 (22.6)	2026 (23.4)	−0.03
Floor/ward	1233 (41.2)	1636 (41.6)	0.01	982 (39.4)	3549 (41.0)	−0.02
Culture data collected						
Blood	2915 (97.4)	3835 (97.5)	−0.01	2413 (96.8)	8362 (96.6)	0.02
Bronchoalveolar lavage	48 (1.6)	59 (1.5)	0	30 (1.2)	113 (1.3)	−0.01
Cerebrospinal fluid	63 (2.1)	87 (2.2)	−0.01	22 (0.9)	78 (0.9)	0
Endotracheal aspirate	69 (2.3)	87 (2.2)	0	60 (2.4)	164 (1.9)	0.03
Pleural fluid	60 (2.0)	87 (2.2)	−0.01	87 (3.5)	216 (2.5)	0.06
Peritoneal fluid	27 (0.9)	35 (0.9)	0	27 (1.1)	104 (1.2)	−0.01
Sputum	428 (14.3)	582 (14.8)	−0.01	386 (15.5)	1307 (15.1)	0.01
Upper respiratory secretion	24 (0.8)	31 (0.8)	0	15 (0.6)	61 (0.7)	−0.02
MRSA swab	1137 (38.0)	1510 (38.4)	−0.01	1050 (42.1)	3523 (40.7)	0.03
Antibiotic treatments						
Anti-MRSA antibiotics						
Vancomycin	2879 (96.2)	3811 (96.9)	−0.04	1892 (75.9)	6553 (75.7)	0
Linezolid	236 (7.9)	264 (6.7)	0.05	105 (4.2)	286 (3.3)	0.05
Clindamycin	111 (3.7)	165 (4.2)	−0.02	52 (2.1)	216 (2.5)	−0.02
Daptomycin	12 (0.4)	16 (0.4)	0.01	32 (1.3)	104 (1.2)	0.01
Other	242 (8.1)	326 (8.3)	−0.01	239 (9.6)	857 (9.9)	−0.01
Anti-PSA antibiotics						
Cefepime	1643 (54.9)	2175 (55.3)	−0.01	1561 (62.6)	5566 (64.3)	−0.03
Piperacillin-tazobactam	982 (32.8)	1282 (32.6)	0	1045 (41.9)	3601 (41.6)	0.01
Carbapenem	168 (5.6)	212 (5.4)	0.01	199 (8.0)	641 (7.4)	0.02
Fluroquinolone	108 (3.6)	146 (3.7)	−0.01	70 (2.8)	251 (2.9)	0
Aztreonam	60 (2.0)	87 (2.2)	−0.01	70 (2.8)	182 (2.1)	0.05
Aminoglycosides	33 (1.1)	51 (1.3)	−0.02	35 (1.4)	95 (1.1)	0.03
Other	30 (1.0)	43 (1.1)	−0.01	30 (1.2)	87 (1.0)	0.02
PSA/MRSA de-escalation						
Continued	1 [Reference]	1 [Reference]	NA	1 [Reference]	1 [Reference]	NA
De-escalated	347 (11.6)	499 (12.7)	−0.04	411 (16.5)	1463 (16.9)	−0.01
Not applicable	1116 (37.3)	1416 (36.0)	0.03	1496 (60.0)	5237 (60.5)	−0.01

Abbreviations: BMI, body mass index; FIO_2_, fractional inspired oxygen; HFNC, high-flow nasal cannula; ICU, intensive care unit; MRSA, methicillin-resistant *Staphylococcus aureus*; NA, not applicable; NIPPV, noninvasive positive pressure ventilation; Pao_2_, partial pressure of arterial oxygen; PSA, *Pseudomonas aeruginosa* (or other resistant gram-negative bacteria); SMD, standardized mean difference.

SI conversion factor: To convert hemoglobin to g/L, multiply by 10; white blood cell count to ×10^9^/L, multiply by 0.001; lactate to mmol/L, multiply by mg/dL; platelet count to ×10^9^/L, multiply by 1; creatinine to μmol/L, multiply by 88.4; bilirubin to μmol/L, multiply by 17.104.

^a^ The other race category included American Indian or Alaskan Native race, Arab and Chaldean ancestry, Asian race, Native Hawaiian or Other Pacific Islander race, and other race.

^b^ Calculated as weight in kilograms divided by height in meters squared.

#### Anti-PSA De-Escalation

Among 36 924 total patients, 18 956 (51.3%) initiated anti-PSA antibiotics on days 1 or 2, of which 12 996 (68.6%) continued receiving anti-PSA antibiotics on day 3 (Figure 1). Of these, 1669 had positive PSA cultures and 178 died on day 3, yielding 11 149 patients for enrollment into the target trial of anti-PSA de-escalation. Of 11 149 patients in the target trial of anti-PSA de-escalation, 5442 (48.1%) had cultures positive for other organisms, 5477 (49.1%) had negative cultures, and 230 (2.1%) had no cultures obtained on days 1 or 2.

A total of 2493 patients (22.4%) in the target trial of anti-PSA de-escalation were de-escalated on day 4. De-escalated patients had a median (IQR) age of 70 (60-79) years and 1314 (55.4%) were male; patients who continued BSA therapy had a median (IQR) age of 70 (60-79) years and 4497 (58.8%) were male (eTable 5 in Supplement 1). The most imbalanced characteristics in the anti-PSA target trial were anti-MRSA continuation, anti-MRSA de-escalation, and genitourinary infection. After weighting, the de-escalated and continued BSA therapy groups were well balanced on propensity scores, patient characteristics (SMD less than 0.10 for all), and treating hospital (Table 1; eFigures 1 and 2 and eTables 5 and 7 in Supplement 1).

#### Variation in De-Escalation Across Hospitals

Across 67 hospitals, there was more than 2-fold variation in the proportion of eligible patients de-escalated from anti-MRSA (median [IQR; range] patients, 42.3% [37.2%-48.9%; 27.3%-61.7%]) and anti-PSA antibiotics (median [IQR; range] patients, 22.3% [19.1%-27.5%; 6.9%-37.7%]) during the study period (eFigures 3 and 4 in Supplement 1).

### Primary Outcomes

**Figure 2.**
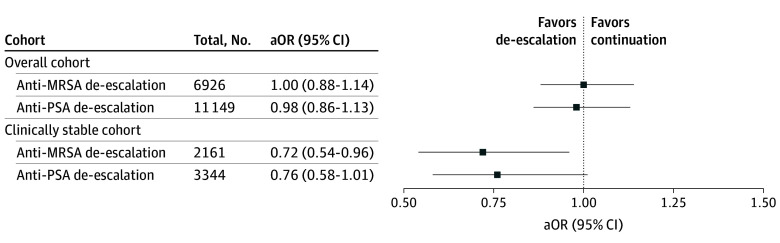
Forest Plot for 90-Day Mortality aOR indicates adjusted odds ratio; MRSA, methicillin-resistant *Staphylococcus aureus*; PSA, *Pseudomonas aeruginosa* (or other resistant gram-negative bacteria).

Primary outcomes are presented in Figure 2, Table 2, and Table 3. In weighted analyses, anti-MRSA and anti-PSA de-escalation were associated with similar 90-day mortality to continued BSA therapy (anti-MRSA: OR, 1.00; 95% CI, 0.88-1.14; anti-PSA: OR, 0.98; 95% CI, 0.86-1.13).

**Table 2.** Secondary and Exploratory Outcomes Associated With Anti–Methicillin-Resistant *Staphylococcus aureus* De-Escalation vs Continuation

Outcome	Patients, No. (%)		Weighted estimate (95% CI)	E-value
	De-escalated therapy (n = 2993)	Continued therapy (n = 3933)		
Primary outcome				
90-d Mortality	858 (28.7)	990 (25.2)	OR, 1.00 (0.88-1.14)	NA
Secondary outcomes				
In-hospital mortality	339 (11.3)	402 (10.2)	OR, 0.87 (0.72-1.05)	NA
30-d Mortality	624 (20.8)	743 (18.9)	OR, 0.92 (0.8-1.06)	NA
In-hospital mortality or hospice discharge	613 (20.5)	753 (19.2)	OR, 0.90 (0.78-1.04)	NA
Days of antibiotic therapy, median (IQR), d	8 (5-13)	10 (7-14)	RR, 0.91 (0.89-0.93)	1.43
Length of hospitalization, median (IQR), d	7 (4-10)	8 (5-12)	RR, 0.88 (0.85-0.92)	1.53
Exploratory outcomes				
90-d Readmission^a^	908 (30.3)	1207 (30.7)	OR, 1.04 (0.92-1.17)	NA
90-d *Clostridioides difficile* infection	18 (0.6)	26 (0.7)	OR, 1.30 (0.47-3.59)	NA

Abbreviations: NA, not applicable; OR, odds ratio; RR, risk ratio.

^a^ Among patients discharged alive and not transferred to another hospital

**Table 3.** Secondary and Exploratory Outcomes Associated With Anti–*Pseudomonas aeruginosa* De-Escalation vs Continuation

Outcome	Patients, No. (%)		Weighted estimate (95% CI)	E-value
	De-escalated therapy (n = 2493)	Continued therapy (n = 8656)		
Primary outcome				
90-d Mortality	635 (25.5)	2655 (30.7)	OR, 0.98 (0.86-1.13)	NA
Secondary outcomes				
In-hospital mortality	225 (9.0)	1050 (12.1)	OR, 1.03 (0.83-1.27)	NA
30-d Mortality	505 (20.3)	1993 (23.0)	OR, 1.10 (0.95-1.28)	NA
In-hospital mortality or hospice discharge	495 (19.9)	1921 (22.2)	OR, 1.11 (0.96-1.29)	NA
Days of antibiotic therapy, median (IQR), d	8 (4-12)	9 (6-13)	RR, 0.91 (0.88-0.93)	1.43
Length of hospitalization, median (IQR), d	5 (3-9)	7 (5-11)	RR, 0.88 (0.80-0.96)	1.53
Exploratory outcomes				
90-d Readmission^a^	731 (29.3)	2770 (32.0)	RR, 0.87 (0.76-0.99)	1.56
90-d *Clostridioides difficile* infection	20 (0.8)	57 (0.7)	OR, 0.96 (0.51-1.80)	NA

Abbreviations: NA, not applicable; OR, odds ratio; RR, risk ratio.

^a^ Among patients discharged alive and not transferred to another hospital.

### Secondary and Exploratory Outcomes

Anti-MRSA and anti-PSA de-escalation were associated with similar in-hospital mortality, 30-day mortality, the composite of in-hospital mortality or hospice discharge, and *C difficile* infection. In weighted analyses, anti-MRSA and anti-PSA de-escalation were associated with fewer days of antibiotics to day 14 (anti-MRSA: RR, 0.91; 95% CI, 0.89-0.93; E-value, 1.43; anti-PSA: RR, 0.91; 95% CI, 0.88-0.93; E-value, 1.43) and shorter length of hospitalization (anti-MRSA: RR, 0.88; 95% CI, 0.85-0.92; E-value, 1.53; anti-PSA: RR, 0.88; 95% CI, 0.80-0.96; E-value, 1.53). Anti-PSA de-escalation (but not anti-MRSA de-escalation) was additionally associated with lower 90-day readmission (RR, 0.87; 95% CI, 0.76-0.96; E-value, 1.56).

### Subgroup Analysis Among Patients With Clinical Stability

Among 6926 patients in the target trial of anti-MRSA de-escalation and 11 149 patients in the target trial of anti-PSA de-escalation, 2161 (31.2%) and 3344 (30.0%), respectively, were clinically stable at time of enrollment and randomization. Of these, 891 (41.2%) and 837 (25.0%) were de-escalated from anti-MRSA and anti-PSA coverage, respectively. Patient characteristics and propensity scores were well balanced in the weighted populations, as shown in eFigures 5 and 6 in Supplement 1. Among clinically stable patients, anti-MRSA de-escalation was associated with lower 90-day mortality (OR, 0.72; 95% CI, 0.54-0.96; E-value, 2.12). The point estimate for anti-PSA de-escalation suggested lower 90-day mortality (OR, 0.76; 95% CI, 0.58-1.01) but was not significant in this smaller subset of patients (Figure 2). Anti-MRSA and anti-PSA de-escalation were both associated with fewer days of antibiotics and shorter length of hospitalization, consistent with the primary analysis (eTables 8 and 9 in Supplement 1).

## Discussion

In this target trial emulation of antibiotic de-escalation in community-onset sepsis, de-escalation from anti-MRSA and anti-PSA coverage were associated with similar mortality but fewer days of antibiotics and shorter length of hospitalization. Among patients who were clinically stable on day 3, de-escalation of anti-MRSA coverage was associated with lower mortality.

Our findings are consistent with several prior observational studies^23,37,38,39,40^ and the recent multicenter SIMPLIFY trial testing de-escalation from antipseudomonal β-lactam to narrower-spectrum therapy in patients with *Enterobacterales* bacteremia.^41^ These prior studies have generally suggested benefit (or no harm) from BSA de-escalation among patients with sepsis. However, some clinicians remain unconvinced,^42^ as underscored by the high prevalence of continued BSA therapy among patients in our study. Those expressing caution about de-escalation often cite a 2014 multicenter nonblinded, noninferiority randomized clinical trial in 9 French intensive care units.^24^ In this trial, patients randomized to antibiotic de-escalation had numerically longer intensive care unit lengths of stay, more superinfections, and longer total durations of antibiotic therapy.^24^ However, these results have been criticized due to nonconsecutive patient enrollment, lack of reporting of antibiotic appropriateness, lack of power necessary to draw conclusions, and substantial group differences that may have driven outcomes.^43^ Increased total antibiotic days in patients undergoing de-escalation has been previously observed.^44,45^ While the etiology of this prolonged antibiotic use is unclear, some have hypothesized that inappropriate clinician counting of antibiotic days received or distrust in the efficacy of narrower-spectrum antimicrobials may contribute.^16,42^ In this study, de-escalation from both anti-MRSA and anti-PSA were associated with fewer days of antibiotic therapy.

Another expressed concern is that improvements in short-term mortality with de-escalation may be due to reverse causation, where improving clinical trajectory increases likelihood of de-escalation, as many observational studies do not adjust for clinical stability.^16^ Given these viewpoints, new data may inform practice. Although observational, by adjusting for predicted mortality on presentation and clinical stability at day 3, we attempted to overcome some barriers encountered by other studies of BSA de-escalation. Furthermore, by requiring that patients remain receiving antibiotics on day 3, we excluded a potentially healthier cohort that is de-escalated earlier in their course. Finally, our findings persist in a cohort of patients clinically stable on day 3. In total, our data lend further evidence suggestive that antibiotic de-escalation is both safe and appropriate in patients hospitalized with community-onset sepsis, bolstering guideline recommendations to do so in clinical practice.^6,7,16^

A second finding of our study was the prevalence and variability of de-escalation in a diverse cohort of hospitals. Fewer than one-half of eligible patients were de-escalated on day 4, and there was more than 2-fold variation in de-escalation of both anti-MRSA and anti-PSA therapy across hospitals. A higher proportion of patients were de-escalated from anti-MRSA therapy compared with anti-PSA therapy. This may reflect both greater clinical certainty and greater ease of de-escalating anti-MRSA therapy. It has become easier to rule out MRSA pneumonia with a nasal swab^46^ and MRSA bacteremia with molecular testing,^47^ while development and dissemination of rapid tests for diagnosis of resistant gram-negative infections have been slower. Furthermore, anti-MRSA therapy can often be discontinued from the antibiotic regimen once MRSA has been ruled out, while anti-PSA therapy often requires transitioning from a broader-spectrum agent to narrower-spectrum agent, which is a more complex decision.

A final, hypothesis-generating finding of our study was that anti-PSA de-escalation was associated with reduced hospital readmission, while anti-MRSA de-escalation was not. Anti-PSA coverage often includes antianaerobic coverage that is deleterious to the gut microbiome and which may increase downstream risk of infection and mortality.^29,48^ One hypothesis for the potential protective effect of de-escalating anti-PSA on hospital readmission is to avoid the deleterious effects of BSA on the gut microbiome and associated downstream risks that may contribute to readmission.^29,48^ Additional prospective randomized studies are needed to further understand the effects of de-escalation of specific classes of antibiotics, as that may drive clinical decision-making.

### Limitations

Our findings should be interpreted in the context of several limitations. First, the study is observational and so cannot exclude the possibility of confounding. We attempted to mitigate confounding by balancing the de-escalated and continued BSA therapy populations on an extensive number of confounders. We also calculated E-values to assess the strength of a hypothetical unmeasured confounder that would be required to negate the observed reductions in days of antibiotic therapy and length of hospitalization. The calculated E-values are small, so it remains possible that residual confounding could negate the observed results even though we adjusted for an extensive number of potential confounders. Second, while we use multiple methods to capture postdischarge mortality, some outcomes may not have been ascertained. Further, readmission and *C difficile* infection may not be fully captured, so we consider these as exploratory outcomes. Third, patients who leave against medical advice or have lengths of stay greater than 120 days are excluded from the HMS-Sepsis registry and so could not be included in this study. However, only a small number of patients are excluded for these reasons. Fourth, we do not have a unique identifier to identify and account for potential repeated hospitalizations within individuals, although abstraction into HMS-Sepsis registry within the prior 90 days is an exclusion criteria, which limits the number of repeated hospitalizations and limits the dependence of repeated admissions.

## Conclusions

In this study, BSA de-escalation on day 4 among patients hospitalized with MDRO-negative community-onset sepsis was associated with similar mortality, fewer days of antibiotic therapy, and shorter length of hospitalization compared with continued BSA therapy. The de-escalated and continued BSA therapy populations were well balanced on detailed baseline characteristics, overcoming limitations of prior observational studies that resulted in doubt regarding the safety of BSA de-escalation.

## Notes

Supplement 1. **eAppendix 1.** Michigan Hospital Medicine Safety (HMS) Collaborative Information **eAppendix 2.** HMS-Sepsis Initiative **eTable 1.** Target Trial Emulation Protocol **eTable 2.** Qualifying Anti-MRSA and Anti-PSA antibiotics **eTable 3.** Variables Used for Balancing the De-Escalated vs Continued Groups in the Target Trial Emulations **eTable 4.** Classification of Site of Infection by CCSR Category **eAppendix 3.** Hospital Medicine Safety (HMS) Registry Definition of Sex, Race, and Ethnicity **eTable 5.** Characteristics of Target Trial Emulation Patients Before Weighting **eTable 6.** Treating Hospital of Target Trial Emulation Patients Before Weighting (Hospitals) **eTable 7.** Treating Hospital of Target Trial Emulation Patients After Weighting (Hospitals) **eFigure 1.** Standardized Mean Difference of Covariates Before (Orange) and After (Blue) Inverse Probability Treatment Weighting, for Patients Continued and De-Escalated From Empiric Anti-MRSA Therapy (Left) and Empiric Anti-PSA Therapy (Right), Target Trial Emulation Cohort **eFigure 2.** Propensity Score Distribution Pre- and Post-Weighting, Primary Analyses **eFigure 3.** Hospital Variation in Anti-MRSA and Anti-PSA De-Escalation **eFigure 4.** Hospital Variation in Anti-MRSA and Anti-PSA De-Escalation, Clinically Stable Subgroup **eFigure 5.** Standardized Mean Difference of Covariates Before (Orange) and After (Blue) Inverse Probability Treatment Weighting, for Patients Continued and De-Escalated From Empiric Anti-MRSA Therapy (Left) and Empiric Anti-PSA Therapy (Right), Clinically Stable Subgroups **eFigure 6.** Propensity Score Distribution Pre- and Post-Weighting, Clinically Stable Subgroups **eTable 8.** Outcomes Associated With Anti-MRSA De-Escalation vs Continuation, Clinically Stable Subgroup **eTable 9.** Outcomes Associated With Anti-PSA De-Escalation vs Continuation, Clinically Stable Subgroup

Supplement 2. Data Sharing Statement
