# Supplementary material for: Antibiotic De-Escalation in Adults Hospitalized for Community-Onset Sepsis
Source: JAMA Intern Med. 2025 Dec 22;186(2):192–202. doi: 10.1001/jamainternmed.2025.6919 (PMC12723592; doi:10.1001/jamainternmed.2025.6919)
Supplement: Supplement 1. — eAppendix 1. Michigan Hospital Medicine Safety (HMS) Collaborative Information eAppendix 2. HMS-Sepsis Initiative eTable 1. Target Trial Emulation Protocol eTable 2. Qualifying Anti-MRSA and Anti-PSA antibiotics eTable 3. Variables Used for Balancing the De-Escalated vs Continued Groups in the Target Trial Emulations eTable 4. Classification of Site of Infection by CCSR Category eAppendix 3. Hospital Medicine Safety (HMS) Registry Definition of Sex, Race, and Ethnicity eTable 5. Characteristics of Target Trial Emulation Patients Before Weighting eTable 6. Treating Hospital of Target Trial Emulation Patients Before Weighting (Hospitals) eTable 7. Treating Hospital of Target Trial Emulation Patients After Weighting (Hospitals) eFigure 1. Standardized Mean Difference of Covariates Before (Orange) and After (Blue) Inverse Probability Treatment Weighting, for Patients Continued and De-Escalated From Empiric Anti-MRSA Therapy (Left) and Empiric Anti-PSA Therapy (Right), Target Trial Emulation Cohort eFigure 2. Propensity Score Distribution Pre- and Post-Weighting, Primary Analyses eFigure 3. Hospital Variation in Anti-MRSA and Anti-PSA De-Escalation eFigure 4. Hospital Variation in Anti-MRSA and Anti-PSA De-Escalation, Clinically Stable Subgroup eFigure 5. Standardized Mean Difference of Covariates Before (Orange) and After (Blue) Inverse Probability Treatment Weighting, for Patients Continued and De-Escalated From Empiric Anti-MRSA Therapy (Left) and Empiric Anti-PSA Therapy (Right), Clinically Stable Subgroups eFigure 6. Propensity Score Distribution Pre- and Post-Weighting, Clinically Stable Subgroups eTable 8. Outcomes Associated With Anti-MRSA De-Escalation vs Continuation, Clinically Stable Subgroup eTable 9. Outcomes Associated With Anti-PSA De-Escalation vs Continuation, Clinically Stable Subgroup [file jamainternmed-e256919-s001.pdf]

Supplemental Online Content

Gupta AB, Heath M, Walzl E, et al. Antibiotic de-escalation in adults hospitalized for community-onset sepsis. *JAMA Intern Med*. Published online December 22, 2025. doi:10.1001/jamainternmed.2025.6919

eAppendix 1. Michigan Hospital Medicine Safety (HMS) Collaborative Information

eAppendix 2. HMS-Sepsis Initiative

eTable 1. Target Trial Emulation Protocol

eTable 2. Qualifying Anti-MRSA and Anti-PSA antibiotics

eTable 3. Variables Used for Balancing the De-Escalated vs Continued Groups in the Target Trial Emulations

eTable 4. Classification of Site of Infection by CCSR Category

eAppendix 3. Hospital Medicine Safety (HMS) Registry Definition of Sex, Race, and Ethnicity

eTable 5. Characteristics of Target Trial Emulation Patients Before Weighting

eTable 6. Treating Hospital of Target Trial Emulation Patients Before Weighting (Hospitals)

eTable 7. Treating Hospital of Target Trial Emulation Patients After Weighting (Hospitals)

eFigure 1. Standardized Mean Difference of Covariates Before (Orange) and After (Blue) Inverse Probability Treatment Weighting, for Patients Continued and De-Escalated From Empiric Anti-MRSA Therapy (Left) and Empiric Anti-PSA Therapy (Right), Target Trial Emulation Cohort

eFigure 2. Propensity Score Distribution Pre- and Post-Weighting, Primary Analyses

eFigure 3. Hospital Variation in Anti-MRSA and Anti-PSA De-Escalation

eFigure 4. Hospital Variation in Anti-MRSA and Anti-PSA De-Escalation, Clinically Stable Subgroup

eFigure 5. Standardized Mean Difference of Covariates Before (Orange) and After (Blue) Inverse Probability Treatment Weighting, for Patients Continued and De-Escalated From Empiric Anti-MRSA Therapy (Left) and Empiric Anti-PSA Therapy (Right), Clinically Stable Subgroups

eFigure 6. Propensity Score Distribution Pre- and Post-Weighting, Clinically Stable Subgroups

eTable 8. Outcomes Associated With Anti-MRSA De-Escalation vs Continuation, Clinically Stable Subgroup

eTable 9. Outcomes Associated With Anti-PSA De-Escalation vs Continuation, Clinically Stable Subgroup

This supplementary material has been provided by the authors to give readers additional information about their work.

© 2025 Gupta AB et al. *JAMA Internal Medicine*.

eAppendix 1. Michigan Hospital Medicine Safety (HMS) Collaborative Information

General Overview
The Hospital Medicine Safety Consortium (HMS) is a Collaborative Quality Initiative (CQI), launched in 2010 with 16 participating hospitals. The goal of HMS is to improve the quality of care for hospitalized medical patients who are at risk for adverse events. By 2022, HMS membership increased to 69 hospital partners across the state of Michigan.

The HMS CQI targets hospitalists, as well as intensivists and emergency medicine physicians. Due to their management of diverse groups of hospitalized patients and close interactions with hospital staff such as sub-specialists, nurses, and pharmacists, hospitalists are ideally positioned to champion quality improvement (QI) efforts with a goal of preventing adverse events in hospitalized patients.

Sponsorship/Overall Funding Model
HMS is one of more than 20 CQIs sponsored by Blue Cross Blue Shield of Michigan (BCBSM) as a part of their Value Partnerships Program. Value Partnerships is a collection of programs from BCBSM, physicians, and hospitals across Michigan that make health care work better for everyone. It's a unique, collaborative model using data collection and sharing of best practices to help health professionals improve patient outcomes.

Value Partnerships improve the quality of care, making it more cost-effective based on the following guiding principles:

- Partnering with providers to deliver clinical programs and aligned provider payments that improve the delivery of high quality, affordable health care
- Designing and executing programs in a customized and collaborative manner, rather than using a one-size-fits-all approach
- Offering a portfolio of interdependent programs that collectively drive outcomes
- Focusing on investments in transformation of care processes
- Recognizing and rewarding the performance of organizations as well as individual providers
- Rewarding improvement to create meaningful incentives for all participants and partner organizations
- Encouraging collaboration among participants

Pay for Performance
BCBSM's Hospital Pay-for-Performance (P4P) program recognizes short term acute-care hospitals for achievements and improvements in quality and population-health management. Specifically related to the CQIs, hospitals can earn up to 40% of their P4P points based on performance. To determine hospital-specific performance, each CQI annually develops a Hospital Performance Index Scorecard to assess a hospitals performance over a given year related to key quality initiatives.
For more detailed information, please visit the BCBSM Value Partnership website.

HMS Leadership & Committees
The work of the collaborative is led by a leadership team within the HMS Coordinating Center and two committees: the executive committee and the data, design, and publication (DDP) committee.

HMS Coordinating Center
Michigan Medicine houses the Coordinating Center for HMS and is responsible for collecting and analyzing comprehensive clinical data from the participating hospitals. It uses these analyses to evaluate and understand current practice, support the implementation of improvement strategies, and

© 2025 Gupta AB et al. *JAMA Internal Medicine*.

to evaluate change over time. The Coordinating Center supports participants in establishing quality improvement goals and assists them in implementing best practices. For additional Coordinating Center details, please visit the HMS website.

Executive Committee
The purpose of the executive committee is to determine the mission and vision of the collaborative, review current performance and address consortium operational issues, identify and discuss implementation strategies, and establish the future course. The executive committee meets once per year – usually in the Fall – in person. The committee members consist of Coordinating Center Leadership, BCBSM leadership, implementation scientists/local quality experts, and leaders from member hospitals.

Data, Design, and Publications (DDP) Committee
The purpose of the HMS Data, Design, and Publications (DDP) Committee is to review and approve data collection methods, performance index measures, programming issues, data analysis methods, quality improvement materials, and the scholarly work of the collaborative. Physician Champions and Quality Improvement Leads/Administrators from all hospitals are invited to participate on this Committee. A clinical data abstractor lead also participates in this Committee as a representative for that role in discussions. The DDP meets monthly via teleconference and 10 to 15 members attend regularly.

Critical Care Steering Committee
The purpose of the Critical Care Steering Committee is to help drive improvement efforts in intensive care units across Michigan. Engage, support, and disseminate HMS critical care-focused scholarly work, and identify opportunities for collaboration across disciplines caring for the hospitalized medical patient (i.e., general medicine, emergency medicine, infectious diseases, pharmacy, vascular access, etc.).

Consortium Building/Recruitment
Eligible hospitals within the State of Michigan are recruited annually. In 2020, HMS became one of seven required CQIs within the BCBSM Value Partnerships portfolio. If a hospital is eligible for participation in a required CQI and, at the time of recruitment (when the coordinating center reaches out to ask a hospital to join), voluntarily elects not to participate, the hospital forfeits the ability to earn the P4P incentive attributed to the CQI. HMS welcomes approximately 10 hospitals to the consortium each year. The below diagram shows the hospital onboarding since the inception of the collaborative. For a complete list of participating hospitals, please visit our website.

Hospital Participation - Eligibility and Expectations
To participate in HMS, a hospital must meet the following eligibility criteria:

- Be enrolled in BCBSM's Participating Hospital Agreement.
- Have a physician/physician group dedicated to the care of patients in the inpatient hospital setting focused on the care of general medical patients including critical care. For example, hospital program, general internist group, etc.
- Have a sufficient inpatient medical discharge volume of at least 25 eligible adult patients (age 18 years or older) per quarter. Eligibility is based on current QI initiatives. All patients do not have to be managed by the hospitalist program.
- Have organizational commitment for active participation.
- Have an onsite physician champion(s) to act as Clinical Champion.
- Engage members of the multidisciplinary team as it relates to collaborative initiatives. For example, infectious diseases (ID), pharmacy, vascular access, interventional radiology, infection prevention, critical care, etc.

© 2025 Gupta AB et al. *JAMA Internal Medicine*.

- Have a QI/administrative lead who will work on the collaborative initiatives.
- Have or hire a clinical data abstractor (many of the medical record based data elements to be collected and the follow-up patient calls will require someone with a clinical / healthcare background). While the Collaborative doesn't require a nurse for the role of the clinical data abstractor, it is highly recommended. More than 95% of the clinical data abstractors are nurses.
- Participate in all initiatives in which the hospital is eligible for participation

Additional Information

For more information about HMS, please visit the HMS website (http://mi-hms.org/) or follow us on twitter (@HMS_MI). For information related to the CQIs, please visit www.valuepartnerships.com.

© 2025 Gupta AB et al. *JAMA Internal Medicine*.

eAppendix 2. HMS-Sepsis Initiative

Data collection for the HMS-Sepsis initiative involves the abstraction of patient data for 18 eligible cases every 2-week abstraction cycle.

Why we require 18 hospitalizations per abstraction period. Hospitals are required to abstract 18 cases per two-week period. Blue Cross Blue Shield of Michigan funds 1 FTE data abstractor at each hospital. We complete time trials each summer to determine the average time to complete data abstractions. We use these data to set abstraction requirements after accounting for vacation and other duties (e.g., participation in collaborative-wide meetings, completion of biannual survey, time for quality improvement activities, etc.).

How hospitals determine which hospitalizations to abstraction. Potentially eligible hospitalizations during the abstraction period are identified at each hospital based on standardized criteria provided to the hospitals (e.g., diagnostic codes, age). Potentially eligible hospitalizations identified by the standardized criteria are then sorted by minute of discharge time-stamp (i.e., a pseudo-random number). Abstractors then review potentially eligible hospitalizations in order until 18 eligible hospitalizations have been identified and abstracted. Approximately one-third of hospitalizations reviewed are ineligible (generally due to lack of acute organ dysfunction on encounter day 1 or 2). Data are entered into the HMS-sepsis registry via a HIPAA-compliant website that has decision support embedded. The website will indicate whether hospitalizations are eligible vs ineligible after initial data are entered, after which abstractors complete the abstraction for eligible hospitalizations or move to the next case in their list. Compliance with this process is audited by the coordinating center to ensure that hospitals are abstracting a random sample of sepsis hospitalizations.

Inclusion criteria (all 3 required)

1. Principal discharge diagnosis code of sepsis, pneumonia, influenza, COVID-19, or respiratory failure (with secondary diagnosis of pneumonia, influenza, or COVID)

   Sepsis: A02.1, A20.7, A22.7, A26.7, A32.7, A39.1, A40.0, A40.1, A40.3, A40.8, A40.9, A41.01, A41.02, A41.1, A41.2, A41.3, A41.4, A41.50, A41.51, A41.52, A41.53, A41.59, A41.81, A41.89, A41.9, A42.7, A54.86, B37.7, R65.20, R65.21

   Influenza:J09*, J10*, J11*

   Pneumonia: J12* (EXCEPT J12.82, which should be included but considered a COVID code), J13*, J14*, J15*, J16*, J17*, J18*, A48.1, J85*

   COVID: J12.82 & U07.1

   Respiratory Failure: J80*, J96.0*, J96.9*

   Additional rules/guidance: Cases with a primary code of Respiratory failure; must have a Pneumonia or Influenza Code as secondary. For a case with a primary code of COVID, Influenza, or Pneumonia, must have one of the following codes as secondary: D65, D69.59, D69.6, F05, G93.40, G93.41, G93.49, I46.8, I46.9, I95.1, I95.89, I95.9, J80, J96.00, J96.01, J96.02, J96.90, J96.91, J96.92, K72.00, K72.01, K72.90, K72.91, K76.2, K76.3, N17.0, N17.1, N17.2, N17.8, N17.9, R03.1, R06.03, R09.2, R40.20, R40.0, R40.1, R57.0, R57.1, R57.8, R57.9, R65.20, R65.21.

© 2025 Gupta AB et al. *JAMA Internal Medicine*.

2. Evidence of infection. Receipt of qualifying antimicrobial within 2 calendar days of presentation, from CDC's adult sepsis event definition, unless primary diagnosis of COVID.
3. Evidence of acute organ dysfunction present within 2 calendar days of presentation. For principal discharge diagnosis of pneumonia, influenza, or COVID, there must be acute respiratory dysfunction.
    - Supplemental oxygen >4L (35% FIO_2) for >2 hours on a given calendar day and no history of chronic respiratory failure (J96.11) or chronic home oxygen
    - Serum Cr >1.2 AND ≥50% increase from baseline (lowest value during hospitalization) AND no end-stage renal dysfunction (N18.6) or stage V chronic kidney disease prior to admission.
    - Platelet count <100 cells/μL AND > 50% decline in platelets from baseline (highest value during hospitalization)
    - Total bilirubin ≥ 2.0 mg/dL AND doubling of total bilirubin from baseline (lowest value during hospitalization)
    - Lactate > 2.0 mmol/L
    - Treatment with intravenous vasopressor (Angiotensin II, Dopamine, Epinephrine, Norepinephrine, Phenylephrine, or Vasopressin) outside of operating room.
    - Clinical documentation of altered mental status on presentation
      INCLUDE: Confusion, lethargy, reports that the patient is acting out of usual character, worsening deviation from baseline mental status, somnolence, comatose state, encephalopathy, unresponsiveness.

Exclusion criteria

- Pregnant patient
- Patient aged <18 years
- Patient transferred from another hospital (ED to ED Transfer is eligible)
- Major surgery within 2 calendar days of admission
- Patient received IV or IM antibiotics in the 24 hours prior to hospital encounter start
- Comfort measure only / hospice within 3 hours of presentation
- Patient left against medical advice (AMA) or refused medical care
- Length of hospitalization >120 days
- Hospitalization is within 90 days of a prior hospitalization by the same patient that is already abstracted into the HMS-Sepsis registry
- Primary discharge diagnosis is COVID, but there is no positive testing for COVID
- Patients with principal diagnosis of pneumonia, influenza, or COVID who are on chronic supplemental oxygen prior to admission

© 2025 Gupta AB et al. *JAMA Internal Medicine*.

eTable 1. Target Trial Emulation Protocol

Protocol Element	Description	Target Trial
Eligibility Criteria	Who is included?	Patients in the HMS-Sepsis registry hospitalized with community-onset sepsis (November 2020 through June 2024) who mt all of the following criteria:<br>1. were treated with anti-MRSA or anti-PSA therapy on encounter day 1 or day 2<br>2. were continued on anti-MRSA or anti-PSA therapy on encounter day 3<br>3. had no positive MRSA or PSA testing on encounter day 1 or day 2<br>4. were alive through encounter day 3.
Treatment Strategies	Which precise treatment strategies or interventions will eligible individuals receive?	De-escalation of Anti-MRSA Target Trial<br>• De-escalation of anti-MRSA therapy (no anti-MRSA therapy on encounter day 4)<br>• Continuation of anti-MRSA therapy (anti-MRSA therapy on encounter day 4)<br><br>De-escalation of Anti-PSA Target Trial<br>• De-escalation of anti-PSA (no anti-PSA therapy on encounter day 4)<br>• Continuation of anti-PSA therapy (anti-PSA therapy on encounter day 4)
Treatment assignment	How will eligible individuals be assigned to the treatment strategies?	Individuals will be assigned to treatment group based on their encounter day 4 antibiotic therapy.
Outcomes	What outcomes will be measured?	• 90-day all-cause mortality (Primary Outcome)<br>• In-hospital mortality (secondary)<br>• 30-day mortality (secondary)<br>• Composite of in-hospital mortality and hospice discharge (secondary)<br>• Days of antibiotic therapy (secondary)<br>• Length of hospitalization (secondary)<br>• 90-day all-cause readmission (exploratory)<br>• C. difficile infection (exploratory)
Causal estimand	Which causal estimand will be estimated?	Intention-to-treat
Start and end of follow-up	When does follow-up start and when does it end?	Starts at randomization (encounter day 3)<br>Ends at death or 90 days post-discharge
Statistical analysis	Which statistical analyses will be used to estimate the causal estimand?	To control for confounding, we balanced the de-escalated and continued populations on baseline characteristics measured at or before enrollment and randomization using inverse probability of treatment weighting (IPTW). The complete list of balancing variables (included in the propensity score model used for IPTW) is presented in **eTable 3**. Prior to proceeding with the analysis, we checked covariate balance and considered variables with SMDs <0.01 to be well-balanced.<br>We then fit:<br>• Hierarchical logistic regression models for binary outcomes, with a random effect for hospital.<br>• Hierarchical Poisson regression models for count outcomes, with a random effect for hospital.<br>All models used IPTW to balance baseline characteristics
Statistical analysis		Patients with clinical stability at day 3 --defined as not receiving vasopressor therapy, not receiving invasive mechanical ventilation, and having no more than one sign of clinical instability (temperature >38 C, oxygen saturation <90% or on supplemental oxygen therapy above their pre-hospital baseline, heart rate >100 beats/minute, respiratory rate >24 breaths/minute, or systolic blood pressure <90 mmHg)

© 2025 Gupta AB et al. *JAMA Internal Medicine*.

eTable 2. Qualifying Anti-MRSA and Anti-PSA antibiotics

**Class**	**Antibiotic**
Anti-MRSA antibiotics	Ceftaroline
	Clindamycin
	Dalbavancin
	Daptomycin
	Delafloxacin
	Doxycycline
	Linezolid
	Minocycline
	Omadacycline
	Ortivancin
	Tedizolid
	Telvancin
	Trimethoprim-sulfamethoxazole (Bactrim)
	Vancomycin
	Amikacin
Anti-PSA antibiotics	Aztreonam
	Cefepime
	Cefiderocol
	Cefoperazone
	Ceftazidime
	Ceftazidime-avibactam
	Ceftolozane-tazobactage
	Ciprofloxacin
	Colistin
	Doripenem
	Gentamycin
	Imipenem
	Imipenem-cilastatin-relebactam
	Levofloxacin
	Meropenem
	Piperacillin-tazobactam (Zosyn)
	Plazomicin
	Polymyxin B
	Tobramycin

© 2025 Gupta AB et al. *JAMA Internal Medicine*.

eTable 3. Variables Used for Balancing the De-Escalated vs Continued Groups in the Target Trial Emulations

Variable	Time of measurement	Functional Form	Imputation*
**Demographics and baseline health information**			
age	admission	Spline (4 knots)	no missingness
male sex at birth	admission	dichotomous	no missingness
race	admission	categorical (Black/African American, White/Caucasian, other)	no missingness
BMI	admission	Spline (5 knots)	CDC averages for height and weight are imputed when missing or if calculated BMI is <10.
Charlson score (range 0-36)	admission	linear	no missingness
Count of functional (IADL) limitations: eating, bathing, dressing, toileting, transferring, and taking medications (range 0-6)	admission	linear	no missingness
Hospitalization in the prior 90 days	admission	dichotomous	no missingness
Admitted from SNF/SAR/LTAC	admission	dichotomous	no missingness
Moderate/severe kidney disease	admission	dichotomous	no missingness
Hypertension	admission	dichotomous	no missingness
Peripheral vascular disease	admission	dichotomous	no missingness
Cardiovascular disease	admission	dichotomous	no missingness
Atrial fibrillation	admission	dichotomous	no missingness
Congestive heart failure / cardiomyopathy	admission	dichotomous	no missingness
Leukemia or lymphoma	admission	dichotomous	no missingness
Metastatic solid tumor	admission	dichotomous	no missingness
Baseline cognitive impairment	admission	dichotomous	no missingness
Moderate/severe liver disease	admission	dichotomous	no missingness
**Early hospitalization data (pre-enrollment into the target trial)**			
Site of infection			
bacteremia	Discharge diagnosis	dichotomous	no missingness
cardiac	Discharge diagnosis	dichotomous	no missingness
CNS	Discharge diagnosis	dichotomous	no missingness
GI	Discharge diagnosis	dichotomous	no missingness
GU	Discharge diagnosis	dichotomous	no missingness
Skin/soft tissue	Discharge diagnosis	dichotomous	no missingness
Respiratory	Discharge diagnosis	dichotomous	no missingness
Other	Discharge diagnosis	dichotomous	no missingness
None specified	Discharge diagnosis	dichotomous	no missingness
Viral sepsis (positive viral testing; negative bacterial testing)	First 2 days	dichotomous	no missingness

© 2025 Gupta AB et al. *JAMA Internal Medicine*.

Predicted 30-day mortality from HMS-sepsis model	First 6 hours	linear	no missingness
Mechanical ventilation	Day 1-3	dichotomous	no missingness
Altered mental status	Day 1-2	dichotomous	no missingness
Dialysis	Day 1-3	dichotomous	no missingness
**Enrollment (Day 3) Physiology and treatments**			
Highest temperature	Day 3	Categorical:<br>0 = <35 C<br>1 = 35-36 C<br>2 = 36.1-37.8 C<br>3 = 37.9-38.0 C<br>4 = 38.1-38.3 C<br>5 = 38.4-39.9 C<br>6 = 40.0+ C	Day 3 values were used, but if day 3 was missing day 2 was used, if day 3 and 2 were missing then day 1 was used, if all 3 were missing then a normal value (2) was imputed
Highest heart rate	Day 3	Categorical:<br>1 = <60<br>2 = 61-90<br>3 = 91-100<br>4 = 101-124<br>5 = >124	Day 3 values were used, but if day 3 was missing day 2 was used, if day 3 and 2 were missing then day 1 was used, if all 3 were missing then a normal value (2) was imputed
Highest respiratory rate	Day 3	Categorical<br>1 = Normal (< 20)<br>2 = Abnormal (20- 21)<br>3 = Abnormal (22 - 24)<br>4 = Abnormal (25 - 30)<br>5 = Abnormal (> 30)	Day 3 values were used, but if day 3 was missing day 2 was used, if day 3 and 2 were missing then day 1 was used, if all 3 were missing then a normal value (1) was imputed
Lowest SBP on day 3	Day 3	Categorical:<br>1 = Abnormal (< 90 mmHg)<br>2 = Abnormal (90-100 mmHg)<br>3 = Normal (101+ mmHg)	Day 3 values were used, but if day 3 was missing day 2 was used, if day 3 and 2 were missing then day 1 was used, if all 3 were missing then a normal value (3) was imputed
Minimum PaO_2/FiO_2 ratio	Day 3	Spline (4 knots)	Impute 300 for patients who had Pulse Ox=96%+ AND FIO2>0.21 and impute 476 for patients with missing Pulse ox and missing Fio2.
Maximum respiratory support	Day 3	Categorical:<br>Room air<br>Conventional (low-flow) oxygen<br>High-flow nasal oxygen<br>NIPPV<br>Invasive mechanical ventilation	Day 3 values were used, but if day 3 was missing day 2 was used, if day 3 and 2 were missing then day 1 was used, if all 3 were missing then a normal value (room air) was imputed
Lowest hemoglobin	Day 3	Spline (5 knots)	Day 3 values were used, but if day 3 was missing day 2 was used, if day 3 and 2 were

© 2025 Gupta AB et al. *JAMA Internal Medicine*.

			missing then day 1 was used, if all 3 were missing then a normal value (7.5) was imputed
Highest WBC	Day 3	Spline (5 knots)	Day 3 values were used, but if day 3 was missing day 2 was used, if day 3 and 2 were missing then day 1 was used, if all 3 were missing then a normal value (15.75 for male; 13.5 for non-male) was imputed
Highest lactate	Day 3	Spline (5 knots)	Day 3 values were used, but if day 3 was missing day 2 was used, if day 3 and 2 were missing then day 1 was used, if all 3 were missing then a normal value (1) was imputed
Lowest platelet	Day 3	Spline (5 knots)	Day 3 values were used, but if day 3 was missing day 2 was used, if day 3 and 2 were missing then day 1 was used, if all 3 were missing then a normal value (300) was imputed
Highest creatinine	Day 3	Spline (5 knots)	Day 3 values were used, but if day 3 was missing day 2 was used, if day 3 and 2 were missing then day 1 was used, if all 3 were missing then a normal value (1) was imputed
Highest bilirubin	Day 3	Continuous (squared)	Day 3 values were used, but if day 3 was missing day 2 was used, if day 3 and 2 were missing then day 1 was used, if all 3 were missing then a normal value (1) was imputed
Highest procalcitonin	Day 3	Spline (5 knots)	Day 3 values were used, but if day 3 was missing day 2 was used, if day 3 and 2 were missing then day 1 was used, if all 3 were missing then a normal value (1) was imputed
Vasopressors	Day 3	dichotomous	no missingness
Highest level of care	Day 3	Categorical: ICU, step-down, ward/floor	no missingness
**Culture data collected**			
Blood	Day 1-3	dichotomous	no missingness
Bronchoalveolar lavage	Day 1-3	dichotomous	no missingness
Cerebrospinal fluid	Day 1-3	dichotomous	no missingness
Endotracheal aspirate	Day 1-3	dichotomous	no missingness
MRSA swab	Day 1-3	dichotomous	no missingness
Pleural fluid	Day 1-3	dichotomous	no missingness
Peritoneal fluid	Day 1-3	dichotomous	no missingness
Sputum	Day 1-3	dichotomous	no missingness

© 2025 Gupta AB et al. *JAMA Internal Medicine*.

Upper respiratory secretion	Day 1-3	dichotomous	no missingness
**Antibiotic co-treatments**			
MRSA BSA: Vancomycin	Day 1-3	dichotomous	no missingness
MRSA BSA: Daptomycin	Day 1-3	dichotomous	no missingness
MRSA BSA: linezolid	Day 1-3	dichotomous	no missingness
MRSA BSA: clindamycin	Day 1-3	dichotomous	no missingness
MRSA BSA: other	Day 1-3	dichotomous	no missingness
PSA BSA: Zosyn	Day 1-3	dichotomous	no missingness
PSA BSA: Cefepime	Day 1-3	dichotomous	no missingness
PSA BSA: Aztreonam	Day 1-3	dichotomous	no missingness
PSA BSA: Fluoroquinolone	Day 1-3	dichotomous	no missingness
PSA BSA: Carbapenem	Day 1-3	dichotomous	no missingness
PSA BSA: Aminoglycoside	Day 1-3	dichotomous	no missingness
PSA BSA: Other	Day 1-3	dichotomous	no missingness
PSA continued on day 4	Day 4	dichotomous	no missingness
PSA de-escalated on day 4	Day 4	dichotomous	no missingness
PSA NA/ unable to de-escalate	Day 4	dichotomous	no missingness
MRSA continued on day 4	Day 4	dichotomous	no missingness
MRSA de-escalated on day 4	Day 4	dichotomous	no missingness
MRSA NA/ unable to de-escalate	Day 4	dichotomous	no missingness

© 2025 Gupta AB et al. *JAMA Internal Medicine*.

eTable 4. Classification of Site of Infection by CCSR Category

Site of infection	DXCCSR*	CCSR Category Description
Cardiac	CIR004	Endocarditis and endocardial disease
Gastrointestinal	DIG001	Intestinal infection
	DIG009	Appendicitis and other appendiceal conditions
	DIG016	Peritonitis and intra-abdominal abscess
	DIG017	Biliary tract disease
	INF005	Foodborne intoxications
Genitourinary	GEN004	Urinary tract infections
	INF010	Sexually transmitted infections (excluding HIV and hepatitis)
	PRG021	Maternal intrauterine infection
	GEN013	Inflammatory conditions of male genital organs
Respiratory	RSP002	Pneumonia (except that caused by tuberculosis)
	RSP003	Influenza
	RSP005	Acute bronchitis
	RSP006	Other specified upper respiratory infections
	RSP007	Other specified and unspecified upper respiratory disease
	INF012	Coronavirus disease – 2019 (COVID-19)
	INF001	Tuberculosis
Central nervous system	NVS001	Meningitis
	NVS002	Encephalitis
	NVS003	Other specified CNS infection and poliomyelitis
	NVS014	CNS abscess
Skin/soft tissue	SKN001	Skin and subcutaneous tissue infections
	MUS001	Infective arthritis
	MUS002	Osteomyelitis
	MUS027	Musculoskeletal abscess
	CIR028	Gangrene
ENT	RSP001	Sinusitis
	RSP004	Acute and chronic tonsillitis
Bacteremia	R78.81	Bacteremia

There is a no CCSR category for bacteremia, so we used the ICD10 diagnosis code.

© 2025 Gupta AB et al. *JAMA Internal Medicine*.

eAppendix 3. Hospital Medicine Safety (HMS) Registry Definition of Sex, Race, and Ethnicity

Gender

Instructions: Review the medical record to determine the gender of the patient.
This is a required field and the form cannot be submitted without an entry in this field.
Select one of the following:

- *"Male"* if the patient is categorized as a man in the medical record.
- *"Female"* if the patient is categorized as a woman in the medical record.
- *"Unknown"* if the patient's gender is unknown.

Ethnicity

Instructions: Review the medical record to determine the patient's ethnicity.
Select one of the following:

- *"Hispanic or Latino"* if patient demographic information indicates patient is of Hispanic descent. The US Census Bureau states that "People who identify their origin as Spanish, Hispanic, or Latino may be of any race."
- *"Non-Hispanic or Latino"* if patient demographic information indicates patient is not of Hispanic descent.
- "*Unknown*" if ethnicity is not reported in the medical record.

Race

Instructions: Review the medical record to determine the patient's race.
Select one of the following:

- *"American Indian or Alaskan Native"* if patient demographic information indicates patient is Native American, American Indian, or Alaska Native.
- *"Arab and Chaldean Ancestries"* if the patient demographic information indicate patient is of Arab or Chaldean Ancestries.
- *"Asian"* if patient demographic information indicates Asian.
- *"Black or African American"* if patient demographic information indicates patient is Black or African American.
- *"Native Hawaiian or Pacific Islander"* if patient demographic information indicates patient is Native Hawaiian or Pacific Islander.
- *"White or Caucasian"* if patient demographic information indicates patient is white or Caucasian.
- *"Other"* if patient demographic information indicates the patient is a race other than what is listed above.
- *"Unknown"* if patient's race is not indicated in the medical record.

© 2025 Gupta AB et al. *JAMA Internal Medicine*.

eTable 5. Characteristics of Target Trial Emulation Patients Before Weighting

	Anti-MRSA de-escalation target trial emulation			Anti-PSA de-escalation target trial emulation		
	De-escalated (N=2,993)	Continued (N=3,933)	SMD	De-escalated (N=2,493)	Continued (N=8.656)	SMD
**Demographics**						
Age, median (IQR)	70 (59, 78)	68 (57, 77)	0.1077	70 (60, 79)	70 (60, 79)	-0.0245
Male sex, %	57.7	58.8	-0.0220	55.4	55.8	-0.0082
Race, %						
White	77.6	78.3	-0.0166	79.9	78.0	0.0458
Black	17.3	17.0	0.0086	14.3	17.0	-0.0760
Other/Unknown	2.9	2.6	0.0227	3.3	2.8	0.0294
**Baseline health information**						
BMI (kg/m^2), median (IQR)	28.3 (23, 34.8)	28.8 (23.5, 35.8)	-0.0892	28 (22.9, 34.5)	27.7 (22.7, 33.9)	0.0227
Charlson comorbidity score, med (IQR)	3 (2, 5)	3 (1, 5)	0.1154	3 (2, 5)	3 (2, 5)	-0.0500
Functional limitations, med (IQR)	0 (0, 5)	0 (0, 5)	-0.0470	0 (0, 5)	0 (0, 5)	-0.1143
Hospitalization in prior 90 days, %	41.1	39.9	0.0241	40.5	44.2	-0.0754
Admitted from SNF/SAR/LTAC, %	16.5	16.6	-0.0042	15.1	18.2	-0.0831
Moderate/severe kidney disease, %	35.7	27.3	0.1805	35.8	35.1	0.0136
Hypertension, %	67.5	68.3	-0.0179	68.6	69.1	-0.0099
Peripheral vascular disease, %	14.1	16.0	-0.0532	13.2	14.1	-0.0253
Cardiovascular disease, %	41.8	40.7	0.0222	42.2	41.8	0.0073
Atrial fibrillation, %	24.8	23.2	0.0363	22.9	25.3	-0.0559
Congestive heart failure, %	26.7	25.6	0.0256	27.1	27.5	-0.0088
Leukemia or lymphoma, %	4.3	4.1	0.0066	4.4	5.0	-0.0303
Metastatic solid tumor, %	11.1	9.0	0.0686	9.9	11.3	-0.0448
Cognitive impairment, %	20.0	19.2	0.0193	19.2	19.8	-0.0156
Moderate/severe liver disease, %	3.8	2.9	0.0523	3.8	3.1	0.0351
Mild liver disease, %	5.6	5.1	0.0232	6.0	5.4	0.0236
**Early hospitalization data (pre-enrollment into the target trial)**						
Site of infection, %						
Bacteremia	0.2	0.0	0.0579	0.2	0.1	0.0398
Cardiac	0.9	1.2	-0.0276	0.6	0.5	0.0106
Central Nervous System	1.3	1.8	-0.0416	0.8	0.5	0.0336
Gastrointestinal	5.5	5.6	-0.0072	7.7	9.2	-0.0561
Genitourinary	14.9	11.5	0.1002	23.3	16.2	0.1796
Skin/soft tissue	16.4	26.9	-0.2560	10.6	12.2	-0.0517
Respiratory	42.8	37.7	0.1047	38.5	44.1	-0.1124
Other specified	0.3	0.4	-0.0120	0.2	0.3	-0.0179
Not identified	17.6	14.8	0.0763	18.1	16.9	0.0317
Viral Sepsis, %	5.2	5.1	0.0069	6.0	6.1	-0.0020
Predicted 30-day mortality, med (IQR)	0.15 (0.06, 0.31)	0.12 (0.05, 0.26)	0.1636	0.14 (0.06, 0.32)	0.16 (0.07, 0.32)	-0.0476
Altered mental status, %	52.4	47.7	0.0944	49.4	48.6	0.0156
Receipt of dialysis, %	6.7	2.5	0.1983	5.8	6.2	-0.0168
Receipt of mechanical ventilation, %	15.4	14.1	0.0355	11.5	14.7	-0.0963
**Enrollment (day 3) physiology and treatments**						
Highest temperature, %						
< 35.0 C	0.1	0.0	0.0384	0.1	0.0	0.0308
35.0-36.0 C	0.4	0.2	0.0464	0.4	0.4	-0.0014
36.1-37.8 C	86.9	84.7	0.0636	87.6	85.9	0.0492
37.9-38.0 C	2.7	3.7	-0.0564	2.8	3.0	-0.0110
38.1 C to 38.3 C	3.4	3.8	-0.0173	3.1	3.4	-0.0163
38.4 C to 39.9 C	5.8	6.8	-0.0436	5.1	6.4	-0.0543
≥40 C	0.5	0.7	-0.0255	0.8	0.8	-0.0001
Highest heart rate, %						
<60	0.4	0.5	-0.0124	0.5	0.6	-0.0045
60-90	32.0	30.5	0.0328	36.9	30.5	0.1350
91 - 100	23.8	22.1	0.0397	22.2	22.3	-0.0036
101 - 124	31.9	34.8	-0.0598	29.9	34.1	-0.0915
≥ 125	11.9	12.2	-0.0090	10.5	12.4	-0.0603
Lowest systolic blood pressure, %						
< 90 mm Hg	23.5	20.3	0.0768	19.4	23.2	-0.0941
90-100 mm Hg	20.7	21.9	-0.0302	19.1	21.9	-0.0689
≥101 mm Hg	55.8	57.8	-0.0391	61.5	54.9	0.1347
Highest respiratory rate, %						

© 2025 Gupta AB et al. *JAMA Internal Medicine*.

<20	27.7	30.3	-0.0572	33.5	28.5	0.1100
20-21	20.5	20.1	0.0113	20.8	19.0	0.0456
22-24	14.5	15.8	-0.0357	13.1	16.2	-0.0893
25-30	18.1	16.9	0.0318	16.0	17.2	-0.0333
>30	19.1	16.9	0.0575	16.6	19.2	-0.0655
Lowest PaO_2/FIO_2 (mm Hg), med (IQR)	232.1 (180.6, 309.5)	261.9 (180.6, 309.5)	-0.0607	250 (183.1, 309.5)	232.1 (171.9, 309.5)	0.1358
Maximum respiratory support, %						
Room air	33.1	37.2	-0.0857	40.0	31.6	0.1748
Invasive mechanical ventilation	15.1	14.3	0.0215	10.2	14.7	-0.1365
NIPPV	6.6	5.9	0.0261	6.0	6.7	-0.0289
HFNC	4.3	3.9	0.0208	3.0	4.5	-0.0771
Low-flow oxygen system	40.9	38.6	0.0466	40.8	42.4	-0.0341
Lowest hemoglobin (g/dL), med (IQR)	10.1 (8.5, 11.7)	10.1 (8.7, 11.7)	-0.0399	10.2 (8.6, 11.7)	9.9 (8.4, 11.4)	0.0216
Highest white blood cell count (cells/µL), med (IQR)	11.4 (7.7, 16.1)	11.2 (8, 16)	0.0003	11.2 (7.7, 15.7)	11.4 (7.8, 16.2)	0.0071
Highest lactate (mmol/L), med (IQR)	1.8 (1.2, 2.7)	1.9 (1.2, 2.7)	0.0277	1.9 (1.3, 2.8)	1.8 (1.2, 2.7)	0.0677
Lowest platelet count (cells/µL), med (IQR)	191 (129, 270)	203 (142, 282)	-0.1105	188 (129, 256)	196 (133, 277)	-0.0949
Highest creatinine (mg/dL), med (IQR)	1 (0.7, 1.6)	0.9 (0.7, 1.2)	0.3127	1 (0.8, 1.7)	1 (0.7, 1.7)	0.0181
Highest bilirubin (mg/dL), med (IQR)	0.7 (0.4, 1)	0.6 (0.4, 1)	0.0695	0.7 (0.4, 1)	0.7 (0.4, 1)	-0.0001
Highest procalcitonin (µg/L), med (IQR)	433	354.6	0.0390	520.5	445.5	0.0328
Vasopressors administered, %	15.6	14.4	0.0325	13.7	16.1	-0.0664
Highest level of care, %						
ICU	36.5	32.7	0.0798	31.6	36.7	-0.1060
Step-down	41.5	42.0	-0.0086	41.2	40.9	0.0051
Floor/Ward	22.0	25.3	-0.0795	27.2	22.4	0.1106
**Culture data collected**						
Blood	97.4	97.5	-0.0058	97.4	96.3	0.0593
Bronchoalveolar lavage	1.8	1.6	0.0177	1.2	1.4	-0.0209
Cerebrospinal fluid	2.2	2.1	0.0060	1.3	0.8	0.0553
Endotracheal aspirate	2.3	2.0	0.0200	1.6	2.0	-0.0353
Pleural fluid	2.0	2.2	-0.0121	1.8	2.7	-0.0548
Peritoneal fluid	1.0	0.8	0.0165	1.1	1.2	-0.0143
Sputum	15.5	13.4	0.0589	13.8	15.4	-0.0459
Upper respiratory secretion	0.6	0.8	-0.0154	0.4	0.8	-0.0504
MRSA swab	45.1	31.3	0.2883	36.2	41.9	-0.1169
**Antibiotic treatments**						
Anti-MRSA antibiotics						
Vancomycin	98.1	96.2	0.1145	74.4	76.1	-0.0390
Linezolid	3.7	8.1	-0.1901	3.0	3.3	-0.0152
Clindamycin	3.2	5.3	-0.1052	2.4	2.5	-0.0046
Daptomycin	0.6	0.2	0.0668	1.0	1.2	-0.0201
Other	9.4	7.2	0.0788	10.7	9.6	0.0347
Anti-PSA antibiotics						
Cefepime	56.4	56.3	0.0009	65.4	64.0	0.0289
Piperacillin-tazobactam	33.9	30.5	0.0723	37.8	42.7	-0.1001
Carbapenem	5.2	5.4	-0.0076	6.4	7.6	-0.0468
Fluroquinolone	3.5	3.8	-0.0131	3.9	2.7	0.0687
Aztreonam	1.5	2.1	-0.0411	1.6	2.2	-0.0409
Aminoglycosides	1.3	1.0	0.0263	1.3	1.0	0.0309
Other	1.0	1.0	0.0011	1.1	1.0	0.0100
PSA/MRSA De-escalation						
Continued	42.6	59.8	-0.3483	6.7	27.1	-0.5654
De-escalated	20.8	4.3	0.5150	24.9	14.7	0.2584
Unable to be De-escalated	36.6	36.0	0.0133	68.3	58.1	0.2125

BMI=body mass index; HFNC=high-flow nasal cannula; ICU=intensive care unit; IQR=interquartile range; LTAC=long-term acute care; MRSA=methicillin resistant *Staphylococcus aureus*; NIPPV=noninvasive positive pressure ventilation; PSA=pseudomonas; SAR=subacute rehabilitation; SNF=skilled nursing facility

© 2025 Gupta AB et al. *JAMA Internal Medicine*.

eTable 6. Treating Hospital of Target Trial Emulation Patients Before Weighting (Hospitals)

	Anti-MRSA de-escalation target trial emulation			Anti-PSA de-escalation target trial emulation		
	De-escalated (N=2,993)	Continued (N=3,933)	SMD	De-escalated (N=2,493)	Continued (N=8.656)	SMD
**Hospitals**						
1	2.4	1.5	0.0675	3.4	2.1	0.0797
2	0.4	0.7	-0.0338	0.5	1.2	-0.0759
3	3.1	3.1	-0.0031	3.0	3.7	-0.0358
4	1.2	1.2	-0.0047	1.6	1.5	0.0023
5	0.1	0.2	-0.0147	0.1	0.1	0.0124
6	1.3	1.7	-0.0378	1.5	1.7	-0.0165
7	2.1	2.4	-0.0249	2.8	2.6	0.0175
8	2.0	2.7	-0.0511	2.7	1.4	0.0941
9	0.3	0.2	0.0189	0.2	0.2	0.0176
10	2.2	3.1	-0.0536	2.2	2.5	-0.0218
11	1.5	0.8	0.0591	1.8	1.3	0.0404
12	3.5	3.2	0.0165	2.0	4.2	-0.1243
13	1.1	1.4	-0.0277	0.9	0.9	0.0060
14	0.8	0.8	0.0007	1.2	1.1	0.0122
15	2.7	3.4	-0.0400	2.2	2.7	-0.0301
16	0.2	0.3	-0.0162	0.1	0.2	-0.0138
17	0.7	1.4	-0.0663	1.0	1.0	-0.0031
18	2.6	4.0	-0.0789	2.2	2.7	-0.0328
19	1.0	0.6	0.0439	0.6	0.8	-0.0274
20	0.3	0.4	-0.0179	0.3	0.2	0.0148
21	1.0	0.5	0.0537	0.8	0.4	0.0549
22	1.1	1.2	-0.0087	2.3	1.6	0.0519
23	0.6	0.7	-0.0106	0.8	1.0	-0.0123
24	1.1	0.9	0.0193	1.3	0.8	0.0492
25	0.9	1.2	-0.0369	0.8	0.9	-0.0100
26	1.1	0.7	0.0383	1.0	0.7	0.0297
27	0.6	0.9	-0.0335	0.3	1.0	-0.0854
28	1.1	0.7	0.0442	0.7	0.6	0.0180
29	2.2	2.8	-0.0364	1.7	2.9	-0.0806
30	0.7	1.4	-0.0705	0.9	0.9	-0.0008
31	4.9	3.3	0.0811	3.9	3.8	0.0074
32	0.4	0.3	0.0155	0.6	0.6	0.0113
33	1.0	1.2	-0.0195	0.5	0.9	-0.0483
34	2.9	2.7	0.0132	3.4	3.7	-0.0153
35	2.5	2.0	0.0350	2.4	2.3	0.0105
36	1.1	0.8	0.0356	1.0	0.7	0.0419
37	1.8	1.8	-0.0045	1.6	1.6	0.0004
38	0.6	0.4	0.0278	1.4	0.8	0.0532
39	1.4	1.6	-0.0155	1.0	1.5	-0.0439
40	2.1	1.7	0.0317	1.2	1.8	-0.0458
41	0.9	0.8	0.0096	0.9	0.7	0.0173
42	0.4	0.7	-0.0499	0.2	0.6	-0.0742
43	2.4	3.1	-0.0404	1.9	2.3	-0.0237
44	1.9	2.0	-0.0082	1.4	1.9	-0.0377
45	0.7	0.7	0.0058	0.8	0.6	0.0271
46	1.4	2.2	-0.0652	2.0	2.3	-0.0208
47	1.6	0.9	0.0618	1.3	1.4	-0.0044
48	1.1	0.9	0.0214	3.2	1.5	0.1096
49	2.2	2.3	-0.0050	1.9	1.8	0.0097
50	1.2	2.1	-0.0694	1.2	1.6	-0.0361
51	1.2	1.3	-0.0160	1.7	1.1	0.0543
52	2.1	2.6	-0.0378	2.3	1.8	0.0386
53	0.7	0.8	-0.0032	1.2	1.1	0.0055
54	1.8	1.9	-0.0064	1.0	1.3	-0.0303
55	2.1	2.1	-0.0021	2.4	2.0	0.0219
56	0.3	0.1	0.0461	0.1	0.2	-0.0216
57	0.1	0.1	0.0066	0.0	0.0	-0.0263
58	4.1	3.5	0.0328	2.5	2.7	-0.0118
59	0.5	0.3	0.0265	0.6	0.6	-0.0081
60	2.2	1.9	0.0192	2.0	2.0	0.0042

© 2025 Gupta AB et al. *JAMA Internal Medicine*.

61	3.1	2.3	0.0491	2.8	2.3	0.0315
62	1.6	2.0	-0.0260	3.4	2.2	0.0722
63	1.0	0.5	0.0608	1.3	0.8	0.0505
64	0.9	0.7	0.0207	1.0	1.1	-0.0059
65	3.1	1.6	0.0993	2.4	3.3	-0.0487
66	2.6	2.0	0.0416	2.0	2.2	-0.0145
67	0.3	0.6	-0.0338	0.4	0.6	-0.0347

© 2025 Gupta AB et al. *JAMA Internal Medicine*.

eTable 7. Treating Hospital of Target Trial Emulation Patients After Weighting (Hospitals)

	Anti-MRSA de-escalation target trial emulation			Anti-PSA de-escalation target trial emulation		
	De-escalated (N=2,993)	Continued (N=3,933)	SMD	De-escalated (N=2,493)	Continued (N=8.656)	SMD
**Hospitals**						
1	0.5	0.6	-0.0021	1.0	1.0	-0.0024
2	3.1	3.2	-0.0048	4.7	3.5	0.0582
3	1.1	1.2	-0.0023	1.8	1.6	0.0194
4	0.1	0.1	0.0068	0.1	0.1	-0.0065
5	1.6	1.6	0.0030	1.6	1.7	-0.0058
6	2.3	2.1	0.0139	3.0	2.7	0.0181
7	2.1	2.3	-0.0127	1.5	1.6	-0.0106
8	0.2	0.2	-0.0011	0.1	0.2	-0.0113
9	2.6	2.6	-0.0004	2.4	2.4	-0.0033
10	1.1	1.0	0.0102	1.5	1.4	0.0053
11	3.3	3.1	0.0155	3.1	3.7	-0.0348
12	1.3	1.2	0.0102	0.8	0.9	-0.0059
13	0.8	0.8	0.0074	1.2	1.1	0.0062
14	2.9	2.9	-0.0026	2.8	2.6	0.0108
15	0.2	0.2	-0.0041	0.2	0.2	0.0016
16	1.1	1.1	-0.0039	1.1	1.0	0.0090
17	3.4	3.5	-0.0059	2.3	2.6	-0.0181
18	0.8	0.8	0.0025	0.7	0.7	-0.0004
19	0.4	0.4	0.0073	0.2	0.2	-0.0053
20	0.7	0.7	0.0034	0.5	0.5	0.0067
21	1.7	1.3	0.0325	1.7	1.8	-0.0022
22	0.7	0.7	0.0044	1.2	0.9	0.0271
23	1.0	1.1	-0.0029	1.0	0.9	0.0092
24	1.0	1.1	-0.0048	0.8	0.9	-0.0103
25	0.8	0.9	-0.0060	0.8	0.8	-0.0013
26	0.8	0.7	0.0056	0.5	0.9	-0.0441
27	0.9	0.7	0.0133	0.6	0.6	-0.0057
28	2.6	2.6	0.0013	2.2	2.6	-0.0291
29	1.2	1.1	0.0124	0.7	0.9	-0.0240
30	4.2	4.5	-0.0123	4.5	3.8	0.0336
31	0.3	0.3	-0.0010	0.6	0.6	0.0026
32	1.1	1.0	0.0047	0.8	0.8	-0.0030
33	2.8	2.8	0.0011	3.8	3.6	0.0126
34	2.3	2.4	-0.0039	2.6	2.3	0.0175
35	0.8	0.8	0.0050	0.5	0.7	-0.0211
36	1.8	1.7	0.0018	1.5	1.6	-0.0031
37	0.5	0.8	-0.0288	1.0	1.0	0.0028
38	1.5	1.6	-0.0132	1.6	1.4	0.0196
39	1.9	1.8	0.0062	1.8	1.6	0.0093
40	0.8	0.8	0.0053	0.6	0.8	-0.0135
41	0.6	0.6	0.0010	0.2	0.5	-0.0519
42	2.9	2.8	0.0051	1.9	2.2	-0.0206
43	2.0	1.9	0.0090	1.5	1.8	-0.0209
44	0.6	0.7	-0.0104	0.6	0.7	-0.0092
45	1.8	1.8	0.0003	2.3	2.2	0.0092
46	1.2	1.1	0.0060	1.1	1.3	-0.0188
47	0.9	1.0	-0.0039	1.9	1.9	0.0023
48	2.0	2.2	-0.0122	1.7	1.8	-0.0044
49	1.6	1.6	-0.0019	1.3	1.5	-0.0114
50	1.4	1.3	0.0025	1.6	1.3	0.0245
51	2.1	2.4	-0.0219	1.5	1.9	-0.0303
52	0.7	0.8	-0.0079	1.1	1.1	-0.0023
53	1.7	1.8	-0.0085	1.3	1.2	0.0084
54	2.3	2.6	-0.0175	2.1	2.1	-0.0024
55	0.2	0.2	0.0028	0.3	0.2	0.0153
56	0.1	0.0	0.0005	0.0	0.0	-0.0232
57	3.8	3.7	0.0066	2.7	2.7	0.0040
58	0.4	0.4	0.0068	0.5	0.6	-0.0179
59	2.0	1.8	0.0139	2.4	2.4	-0.0052
60	1.9	1.8	0.0081	1.9	2.0	-0.0078

© 2025 Gupta AB et al. *JAMA Internal Medicine*.

61	2.8	2.9	-0.0066	2.6	2.5	0.0066
62	1.9	2.1	-0.0192	2.6	2.4	0.0077
63	0.7	0.8	-0.0156	0.9	0.9	0.0053
64	0.7	0.8	-0.0029	1.1	1.0	0.0029
65	2.3	2.2	0.0108	3.2	3.1	0.0046
66	2.3	2.2	0.0115	2.0	2.1	-0.0114
67	0.4	0.4	-0.0078	0.5	0.5	-0.0022

© 2025 Gupta AB et al. *JAMA Internal Medicine*.

eFigure 1. Standardized Mean Difference of Covariates Before (Orange) and After (Blue) Inverse Probability Treatment Weighting, for Patients Continued and De-Escalated From Empiric Anti-MRSA Therapy (Left) and Empiric Anti-PSA Therapy (Right), Target Trial Emulation Cohort

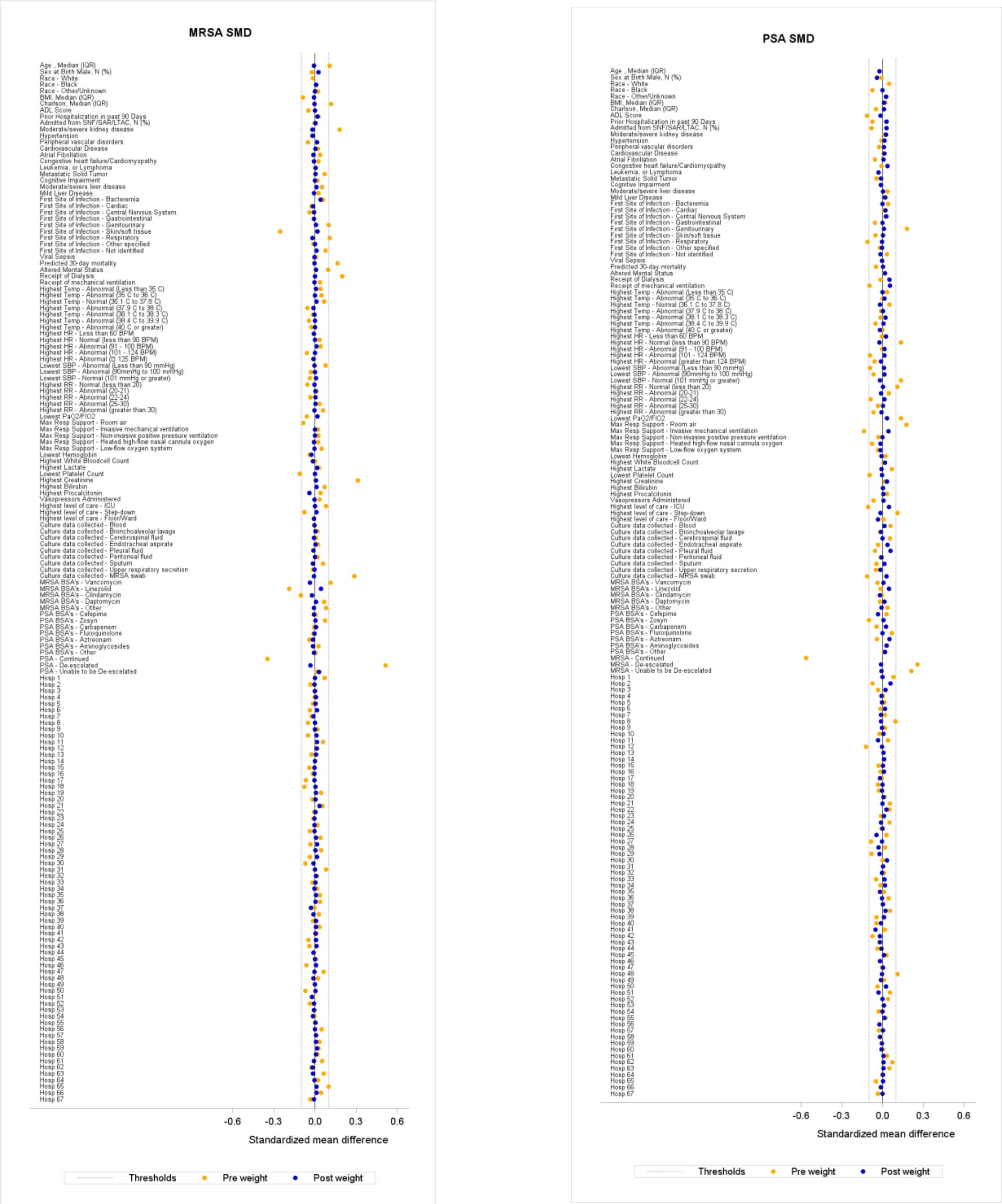

Positive SMDs mean the characteristics is more common in the de-escalated group.
Orange=pre-weighted standardized mean difference (SMD); Blue=post-weighted SMD
Legend: MRSA=*Methicillin-Resistant Staphylococcus* Aureus; PSA=*Pseudomonas Aeruginosa* and other resistant gram negatives; BMI=body mass index; Elevated temperature= >38C, elevated heart rate= pulse >100 beats/minute; increased RR=>24 breaths/minute; SYS BP=systolic blood pressure; ventilation=mechanical ventilation. Elevated temperature, new O2 requirement, elevated heart rate, increased RR, SYS BP less 90, vasopressors, and ventilation were measured on day 3.

© 2025 Gupta AB et al. *JAMA Internal Medicine*.

eFigure 2. Propensity Score Distribution Pre- and Post-Weighting, Primary Analyses

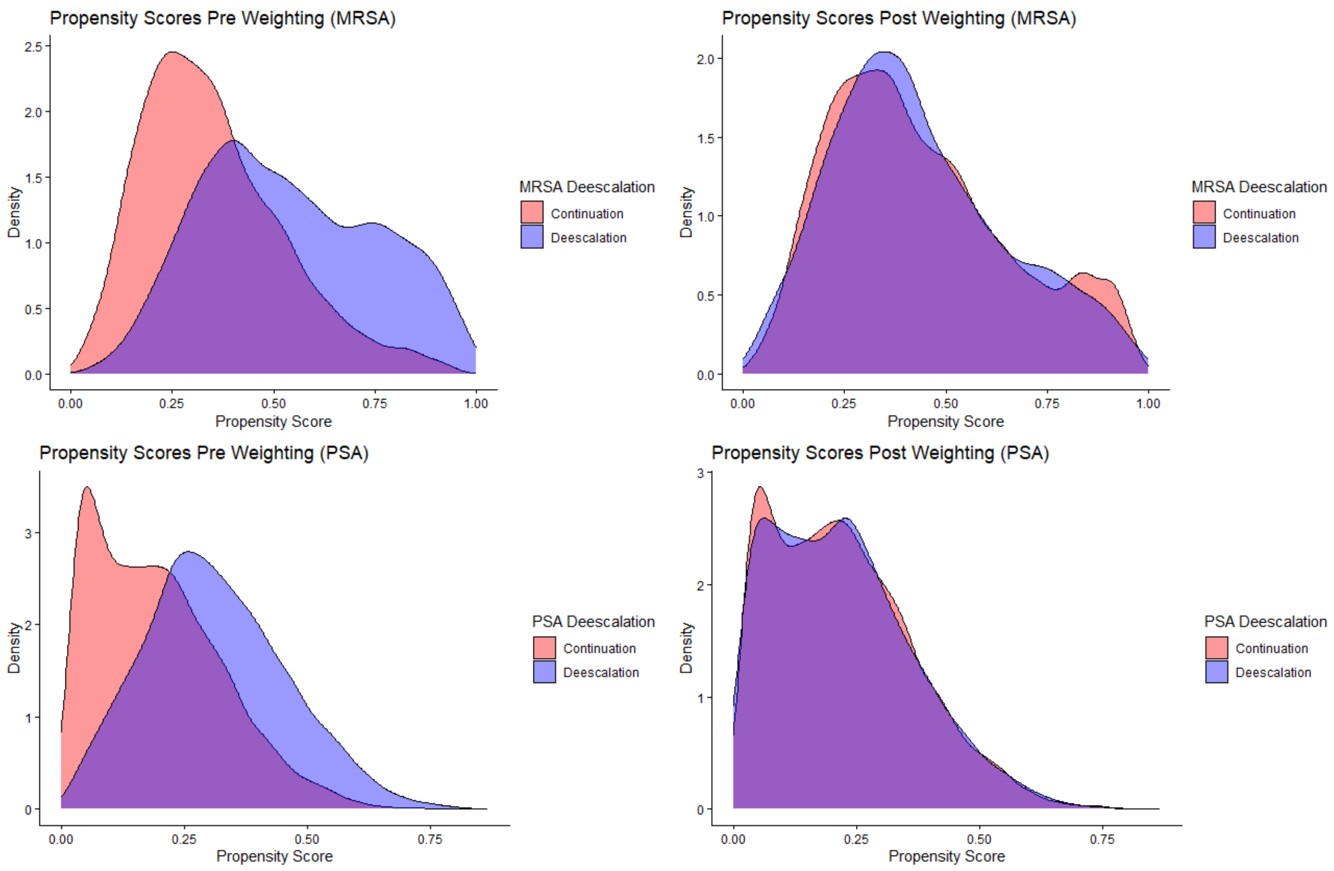

© 2025 Gupta AB et al. *JAMA Internal Medicine*.

eFigure 3. Hospital Variation in Anti-MRSA and Anti-PSA De-Escalation

3a: Hospital variation in anti-MRSA de-escalation

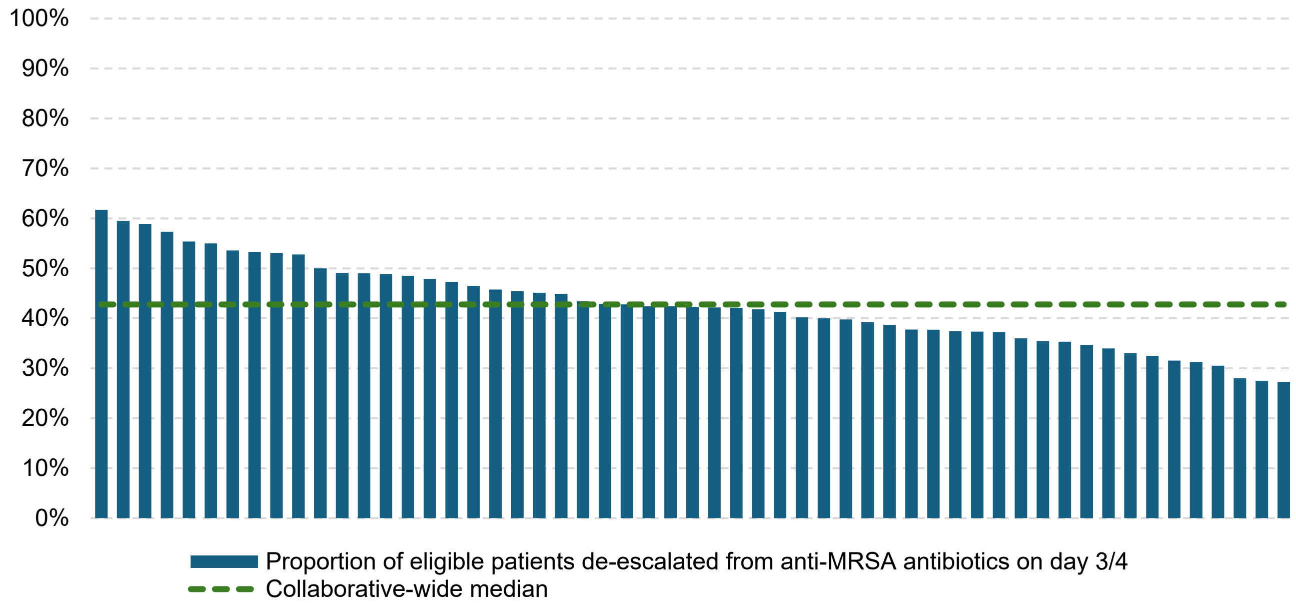

3b: Hospital variation in anti-PSA de-escalation

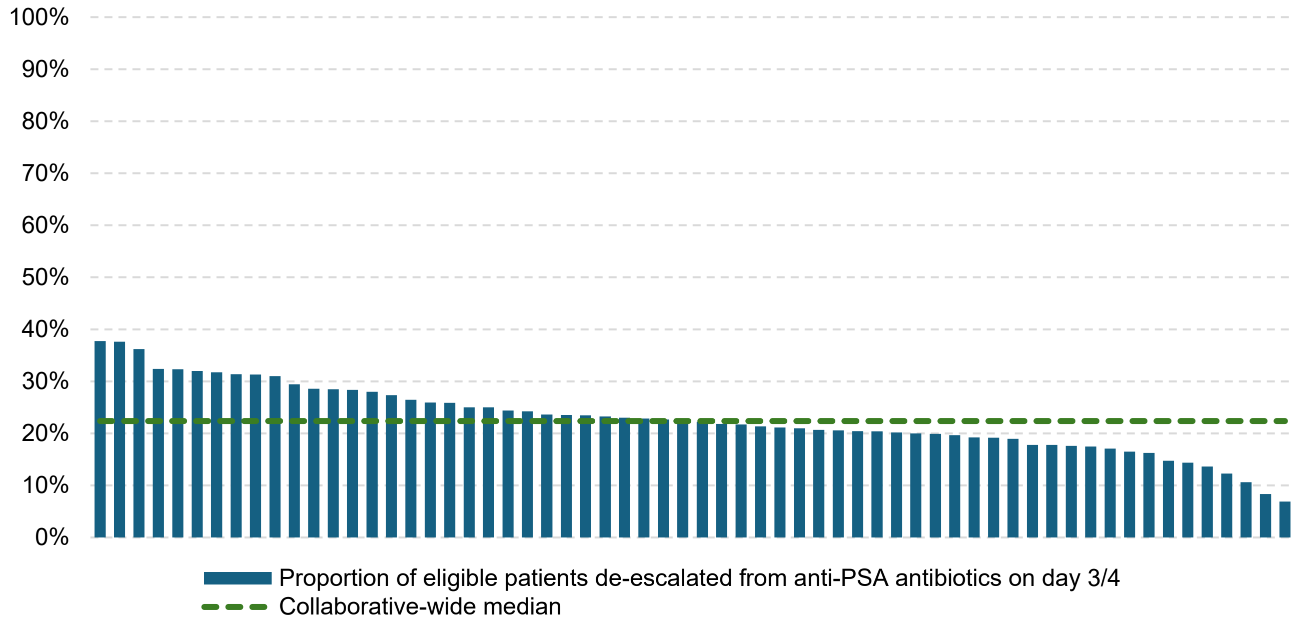

Legend: Each bar reflects one hospital. Hospitals with fewer than 25 eligible patients were suppressed.

© 2025 Gupta AB et al. *JAMA Internal Medicine*.

eFigure 4. Hospital Variation in Anti-MRSA and Anti-PSA De-Escalation, Clinically Stable Subgroup

4a: Hospital variation in anti-MRSA de-escalation, clinically stable subgroup

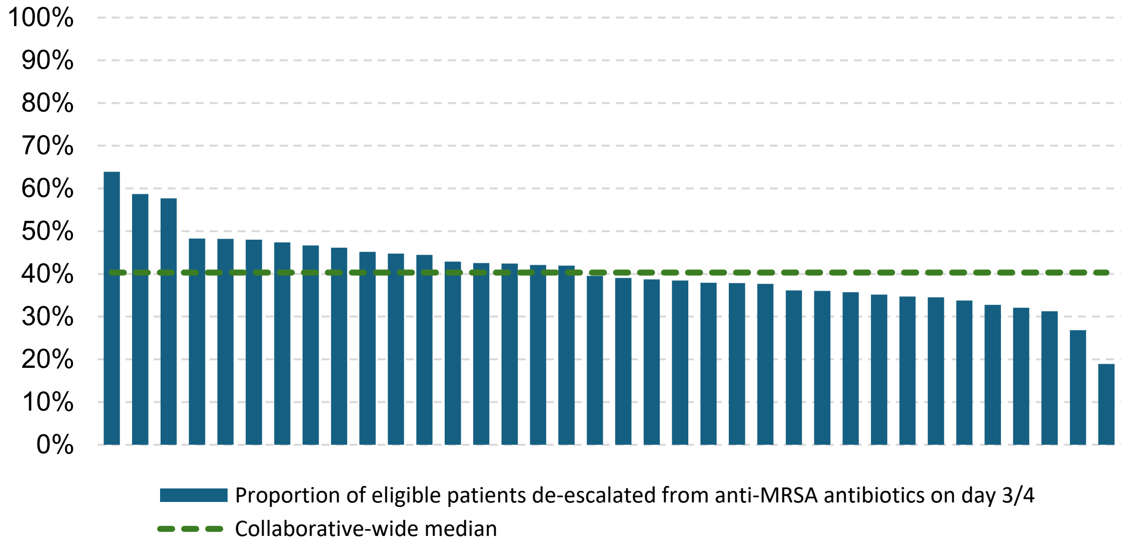

4b: Hospital variation in anti-PSA de-escalation, clinically stable subgroup

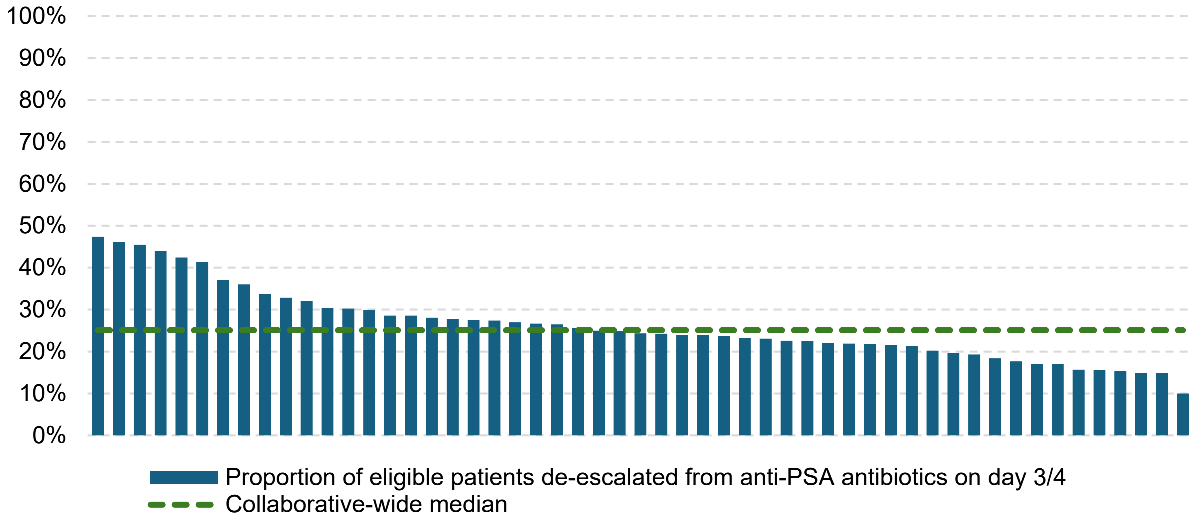

Legend: Each bar reflects one hospital. Hospitals with fewer than 25 eligible patients were suppressed.

© 2025 Gupta AB et al. *JAMA Internal Medicine*.

eFigure 5. Standardized Mean Difference of Covariates Before (Orange) and After (Blue) Inverse Probability Treatment Weighting, for Patients Continued and De-Escalated From Empiric Anti-MRSA Therapy (Left) and Empiric Anti-PSA Therapy (Right), Clinically Stable Subgroups

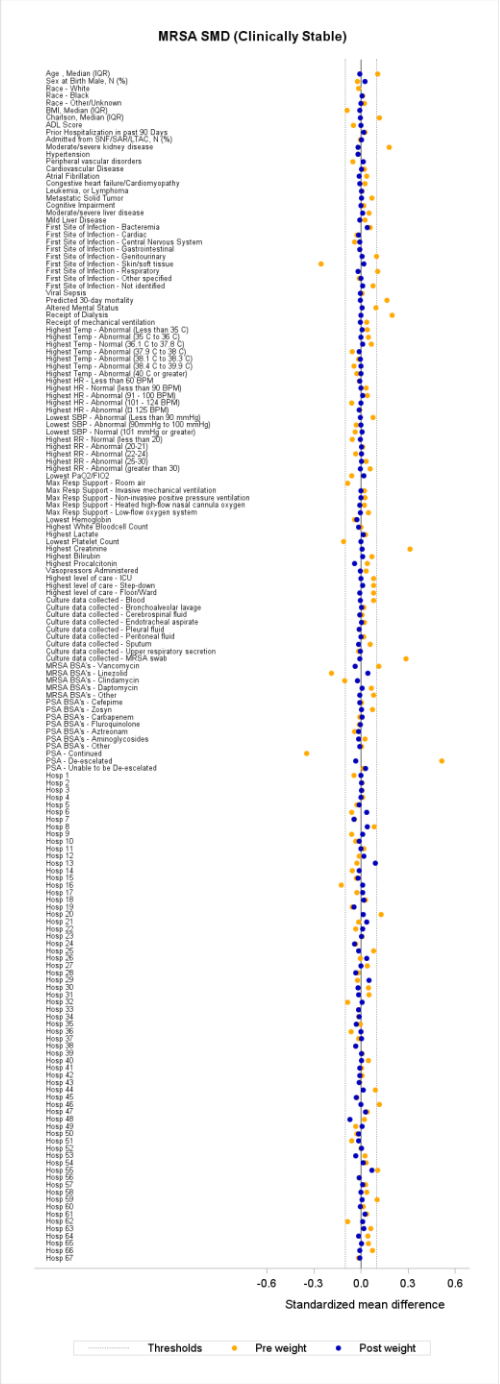

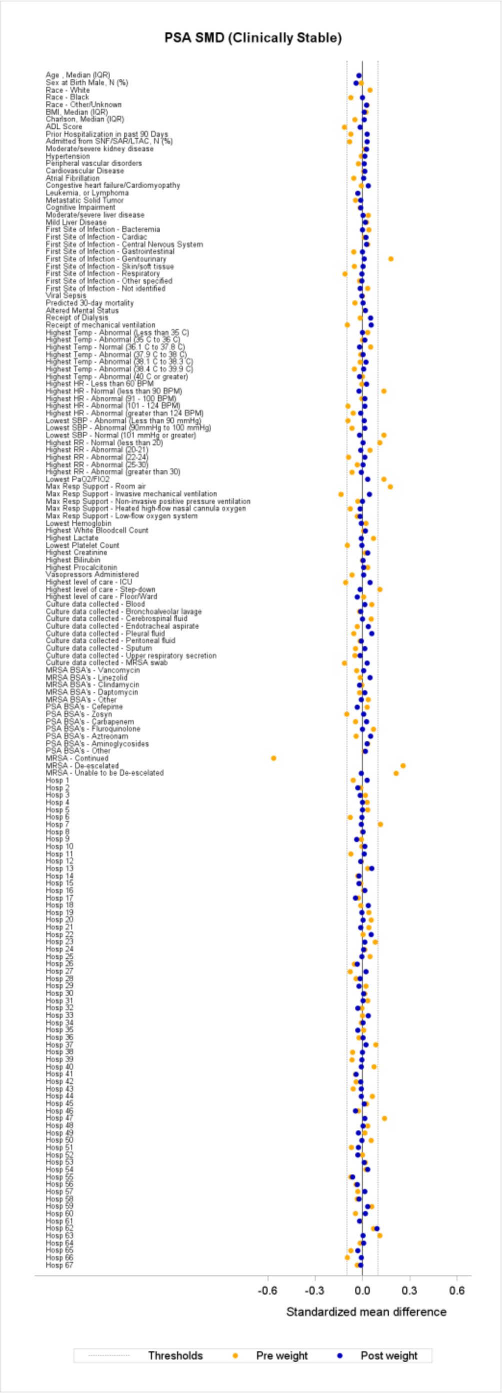

Positive SMDs mean the characteristics is more common in the de-escalated group.
Orange=pre-weighted standardized mean difference (SMD); Blue=post-weighted SMD
Legend: MRSA=*Methicillin-Resistant Staphylococcus* Aureus; PSA=*Pseudomonas Aeruginosa* and other resistant gram negatives; BMI=body mass index; Elevated temperature= >38C, elevated heart rate= pulse >100 beats/minute; increased RR=>24 breaths/minute; SYS BP=systolic blood pressure; ventilation=mechanical ventilation. Elevated temperature, new O2 requirement, elevated heart rate, increased RR, SYS BP less 90, vasopressors, and ventilation were measured on day 3.

© 2025 Gupta AB et al. *JAMA Internal Medicine*.

eFigure 6. Propensity Score Distribution Pre- and Post-Weighting, Clinically Stable Subgroups

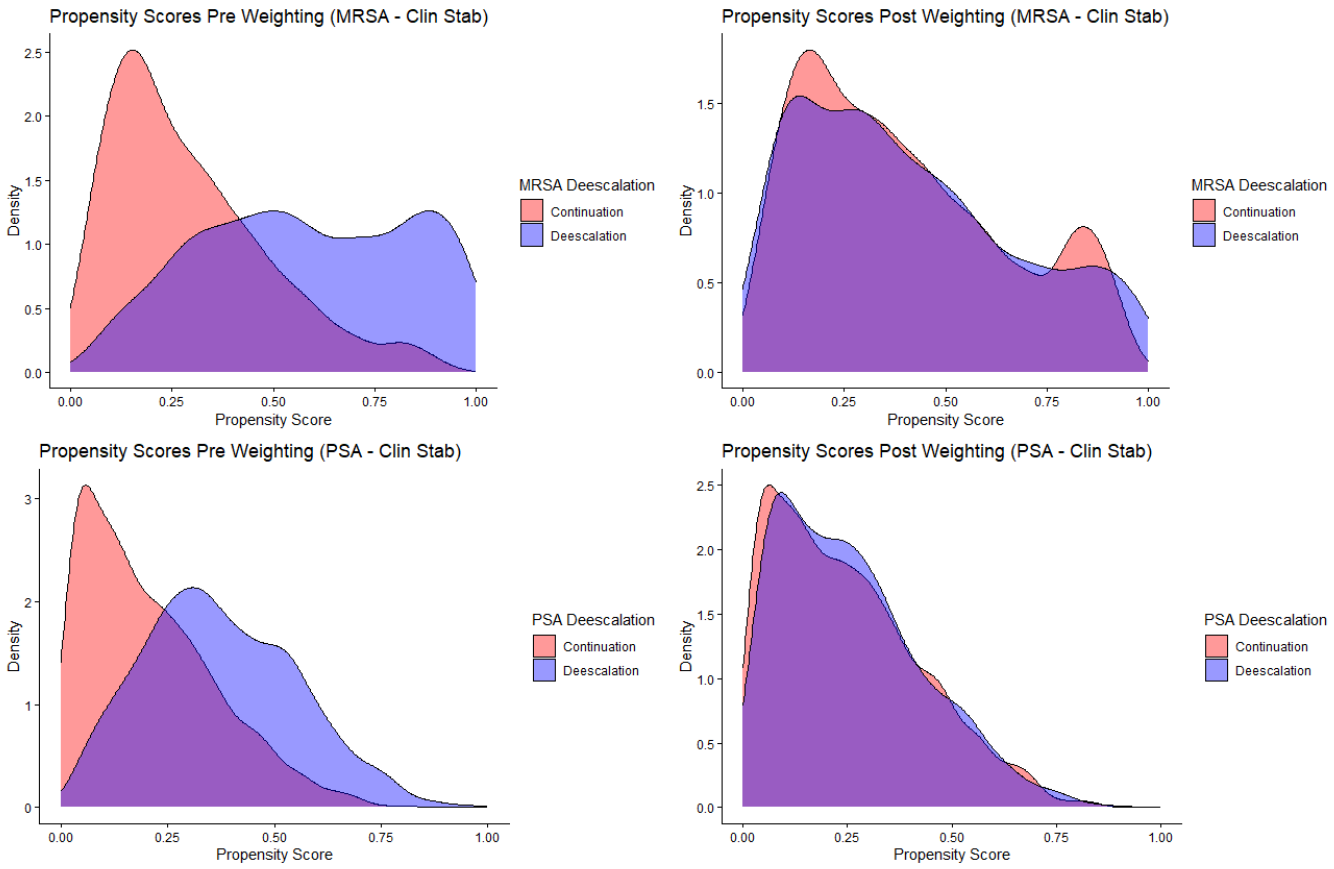

© 2025 Gupta AB et al. *JAMA Internal Medicine*.

eTable 8. Outcomes Associated With Anti-MRSA De-Escalation vs Continuation, Clinically Stable Subgroup

Outcomes	Among N=891 De-escalated, N (%)	Among N=1,270 Continued, N (%)	Odds ratio* in weighted analysis	p	E-value
**Primary outcome**					
90-day mortality	150 (16.8)	209 (16.5)	0.72 (0.54, 0.96)	0.0239	2.124
**Secondary outcomes**					
In-hospital mortality	38 (4.3)	46 (3.6)	0.74 (0.43, 1.25)	0.2546	-
30-day Mortality	90 (10.1)	137 (10.8)	0.65 (0.46, 0.92)	0.0151	2.449
In-hospital mortality or hospice discharge	94 (10.6)	137 (10.8)	0.80 (0.56, 1.14)	0.2099	-
Days of antibiotic therapy	9 (6, 13)	11 (7, 14)	0.89 (0.85, 0.93)	<.0001	1.496
Length of hospitalization, days	5 (3, 8)	6.5 (5, 10)	0.82 (0.74, 0.90)	<.0001	1.737
**Exploratory outcomes**					
90-day readmission[a]	274 (30.8)	407 (32.1)	0.99 (0.76, 1.27)	0.9102	-
90-day *C. difficile* infection	6 (0.7)	7 (0.6)	4.24 (0.88, 20.42)	0.0719	-

[a]Among patients discharged alive and not transferred to another hospital
*The estimates presented for days of antibiotic therapy and length of hospitalization are Risk Ratios with 95% confidence intervals.
Abbreviations: CI: confidence interval; MRSA: methicillin-resistant *Staphylococcus aureus*

© 2025 Gupta AB et al. *JAMA Internal Medicine*.

eTable 9. Outcomes Associated With Anti-PSA De-Escalation vs Continuation, Clinically Stable Subgroup

Outcomes	Among N=837 De-escalated, N (%)	Among N=2,507 Continued, N (%)	Odds ratio* in weighted analysis	p	E-value
**Primary outcome**					
90-day mortality	115 (13.7)	519 (20.7)	0.76 (0.58, 1.01)	0.0556	-
**Secondary outcomes**					
In-hospital mortality	30 (3.6)	107 (4.3)	1.24 (0.74, 2.09)	0.4133	-
30-day Mortality	79 (9.4)	339 (13.5)	0.92 (0.66, 1.28)	0.6203	-
In-hospital mortality or hospice discharge	80 (9.6)	312 (12.5)	1.00 (0.72, 1.39)	0.9868	-
Days of antibiotic therapy	8 (5, 12)	9 (6, 14)	0.92 (0.88, 0.97)	0.0005	1.394
Length of hospitalization, days	4 (3, 6)	6 (4, 9)	0.75 (0.69, 0.81)	<.0001	2.000
**Exploratory outcomes**					
90-day readmission[a]	271 (32.4)	859 (34.3)	0.96 (0.78, 1.19)	0.7225	-
90-day *C. difficile* infection	8 (1.0)	21 (0.8)	0.72 (0.30, 1.71)	0.4578	-

[a]Among patients discharged alive and not transferred to another hospital
*The estimates presented for days of antibiotic therapy and length of hospitalization are Risk Ratios with 95% confidence intervals.
Abbreviations: CI: confidence interval; PSA – *pseudomonas aeruginosa and other resistant gram-negatives*

© 2025 Gupta AB et al. *JAMA Internal Medicine*.

© 2025 Gupta AB et al. *JAMA Internal Medicine*.
