# Supplementary material for: Antibiotic De-Escalation in Adults Hospitalized for Community-Onset Sepsis
Source: JAMA Intern Med. 2025 Dec 22;186(2):192–202. doi: 10.1001/jamainternmed.2025.6919 (PMC12723592; doi:10.1001/jamainternmed.2025.6919)
Supplement: Supplement 2. — Data Sharing Statement [file jamainternmed-e256919-s002.pdf]

Data Sharing Statement

Gupta. Antibiotic De-Escalation in Adults Hospitalized for Community-Onset Sepsis. *JAMA Intern Med*. Published December 22, 2025. doi:10.1001/jamainternmed.2025.6919

Data
Data available: No

Additional Information
Explanation for why data not available: As our initiative is a collaborative of hospitals in Michigan focusing on improving the quality of care for hospitalized patients with sepsis, we utilized the existing infrastructure of the Michigan Hospital Medicine Safety (HMS) Consortium. The data use agreements among the participating hospitals prohibit us from sharing our registry data outside of the Coordinating Center, given hospital specific performance is directly identifiable.
